# Synergistic Approaches in PFAS Remediation: Outlook for Sustainable *In Situ* Mitigation by Combined Adsorption and Photocatalysis

**DOI:** 10.1021/acs.est.6c12980

**Published:** 2026-10-06

**Authors:** Surabhi Sreenitha Raj, Jingqian Chen, Oscar Cabezuelo, Tetyana M. Budnyak

**Affiliations:** † Department of Earth Sciences, 8097 Uppsala University, Villavägen 16, 75236 Uppsala, Sweden; ‡ Wallenberg Initiative Materials Science for Sustainability (WISE), Department of Earth Sciences, Uppsala University, Villavägen 16, 75236 Uppsala, Sweden

**Keywords:** Per- and polyfluoroalkyl substances − PFAS, adsorption, photocatalysis, biopolymers, LCA, AI

## Abstract

Per- and polyfluoroalkyl substances (PFAS) are persistent organic pollutants with widespread environmental occurrence. Their toxicity and resistance to degradation pose serious risks to ecosystems and human health. While existing separation (e.g., adsorption, filtration) and degradation (e.g., photochemical, electrochemical, thermal) technologies can achieve partial PFAS removal, limitations such as high energy demand, poor cost-efficiency, and harsh operating conditions hinder their *in situ* application. In this context, a synergistic approach, a system in which adsorption and degradation are functionally coupled rather than applied as independent sequential unit operations, has emerged as a promising route. This review critically examines the coupling of biopolymer-based adsorption and photocatalytic degradation as a sustainable and effective solution for PFAS remediation. Biopolymers such as lignin, chitosan, and cellulose offer a renewable alternative to conventional sorbents, exhibiting adsorption capacities of ∼50–500 mg g^–1^ and beyond in advanced systems and removal efficiencies of up to 99% in dilute conditions, while photocatalytic platforms, such as semiconductor-based systems, reach ∼32–100% degradation efficiencies, with up to 92% defluorination in optimized systems. When combined functionally, these systems enhance PFAS degradation through interfacial preconcentration and reduce secondary waste. The review critically evaluates recent advances in material development, mechanistic understanding, and system integration, alongside the roles of Life Cycle Assessment and Artificial Intelligence/Machine Learning, as complementary tools guiding the design and deployment of sustainable PFAS-remediation strategies.

## Graphical abstract

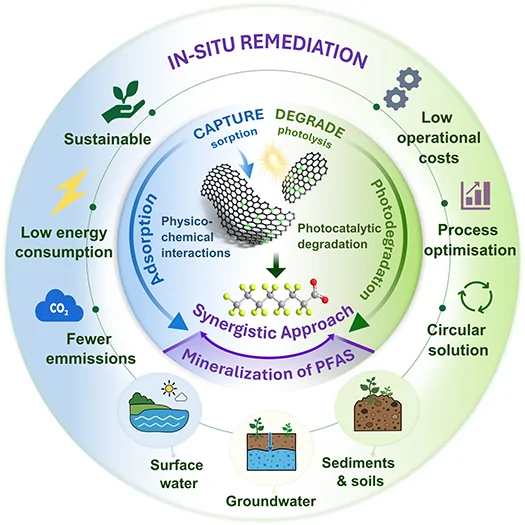

## 1 Introduction

Per- and polyfluoroalkyl substances (PFAS), commonly known as “forever chemicals”, are synthetic organic compounds consisting of short or long carbon chains in which hydrogen atoms are partially or fully substituted by fluorine. Their remarkable stability stems from the strength of the carbon–fluorine (C–F) bond, one of the strongest in organic chemistry with bond dissociation energies typically reported in the range ≈485–540 kJ mol^–1^ depending on the fluorinated environment, which gives PFAS exceptional thermal and chemical resistance. Their hydrophobic and oleophobic properties have led to the extensive use of PFAS in a wide range of industrial and consumer applications, including polymer manufacturing and nonstick cookware,^1^ waterproof materials,^2^ firefighting foams, and electronic components.^3–5^ As a result of their widespread application and exceptional stability, PFAS have become ubiquitous pollutants, detected in soil, groundwater, drinking water, surface water, air, food, and even indoor dust.^6–9^

PFAS mobility and environmental fate are largely influenced by their physicochemical characteristics. Volatile PFAS can undergo long-range atmospheric transport and deposit via wet or dry deposition.^10^ Long-chain PFAS tend to sorb to soil and organic matter, while short-chain PFAS can migrate more easily between environmental phases, hampering remediation efforts.^11–13^ Additionally, PFAS have been found adsorbed on microplastics,^14^ which degrade into particles that release greenhouse gases, contributing indirectly to climate change and leading to a feedback loop of environmental persistence, ecosystem disruption, and increased chemical burden.^15^ Notably, Hayley Hung et al.^16^ reported the detection of PFAS contamination in the Arctic, transported by sea-spray aerosols.

Structurally, PFAS are classified into two broad categories: nonpolymeric and polymeric. Nonpolymeric PFAS include perfluoroalkyl acids (PFAAs), such as Perfluorocarboxylic acids (PFCAs, C_n_F_2__n+1_COOH) and Perfluorosulfonic acids (PFSAs, C_n_F_2__n+1_SO_3_H). Polymeric PFAS comprise high molecular weight substances like fluoropolymers (e.g., polytetrafluoroethylene, PTFE, commonly known as Teflon) and side-chain fluorinated polymers. This classification reflects differences in molecular structure, environmental behavior, and the potential for transformation into persistent perfluoroalkyl acids.^17^ Among these, Perfluorooctanoic Acid (PFOA) and Perfluoro­octanesulfonic Acid (PFOS) remain the most widely produced and detected PFAS.^3,17–19^

A growing body of evidence highlights the toxicological risks associated with PFAS exposure. These compounds have been linked to increased cancer risk, reduced vaccine efficacy, cardiovascular and renal dysfunction, and female infertility.^20–24^ Measured concentrations in human serum from 344 residents affected by drinking-water contamination showed median serum levels of 8.6 ng mL^–1^ for PFOS and 4.3 ng mL^–1^ for PFOA, values that were substantially higher than the reporting limits (0.1–0.5 ng mL^–1^), indicating robust quantification of these compounds.^25^ In addition, organ accumulation has been reported, with mean PFOS concentrations of 6.3 ng g^–1^ in the liver and 3.0 ng g^–1^ in the lungs measured in forensic autopsy samples representative of background exposure, along with trace detection in brain tissue.^26^

Due to their persistence and potential harm, PFAS pose a significant challenge to public health and environmental safety. Regulatory efforts have intensified in recent years. The European Union, for instance, is shifting from substance-specific to group-based restrictions under the Registration, Evaluation, Authorization and Restriction of Chemicals (REACH) framework. A 2023 proposal aims to ban all nonessential uses of PFAS, with enforcement expected by 2026.^27^ The EU Drinking Water Directive (2020/2184) sets a maximum concentration of 100 ng L^–1^ for the sum of 20 PFAS. Several member states have adopted stricter standards; Sweden, for example, limits total PFAS to 100 ng L^–1^ and four specific PFAS – PFOA, PFOS, Perfluorononanoic acid (PFNA) and Perfluorohexanesulfonic acid (PFHxS) to 4 ng L^–1^.^28^ The World Health Organization (WHO) has also acknowledged the carcinogenic potential of PFOA and PFOS, recommending drinking water limits of 100 ng L^–1^ each, and 500 ng L^–1^ for a mixture of 29 PFAS, depending on the treatability by available technologies.^29,30^ The global PFAS crisis directly hinders progress toward several United Nations Sustainable Development Goals (SDGs), notably SDG 3 (Good Health and Well-being), SDG 6 (Clean Water and Sanitation), and SDG 12 (Responsible Consumption and Production).^31,32^ As regulatory thresholds become stricter, the demand for effective, scalable remediation strategies is growing.

Current approaches for PFAS remediation fall into two main categories: physical separation and degradation. Physical separation methods employ materials such as ion-exchange resins,^33,34^ activated carbon (AC),^35,36^ and biobased materials,^37,38^ to remove PFAS from aqueous systems. For example, ion-exchange resins have achieved approximately 55% removal of PFOA and 85% removal of PFOS in spiked groundwater,^34^ and activated carbons removing ∼50% of PFAS from secondary treated municipal wastewater effluent.^36^ Biobased sorbents have also shown promise, achieving approximately ∼80% PFOS removal within 20 min in natural-water-like systems.^38^ However, these materials merely concentrate contaminants without destroying them. On the other hand, degradation-based approaches aim to mineralize PFAS into smaller molecules. Among these, studies on electrochemical oxidation,^39^ advanced oxidation/reduction,^40^ and photochemical degradation^41–44^ have been extensively investigated. Field-scale foam fractionation achieved ∼50% total PFAS removal^40^ and, photochemical treatments have reported removals ranging from ∼16% after 4 h using photocatalytic ozonation^41^ to >80% degradation of long-chain PFAS in landfill leachate after 24 h of photocatalysis.^43^

In response, research is increasingly focused on sustainable alternatives. Biopolymers have emerged as promising candidates due to their renewability, biocompatibility, and low carbon footprint. Similarly, heterogeneous photocatalysis has shown strong potential for mineralizing organic molecules,^45–47^ with its environmental friendliness, energy efficiency, and operational versatility.^48,49^ The recently proposed integration of multiple treatment methods into a unified “treatment train” approach and individual treatment strategies working in a sequence, can overcome the limitations of individual technologies and improve overall performance.^50^ However, despite growing interest, limited attention has been paid to the *in situ* application of biopolymer adsorbents, photocatalysis, and synergistic treatment systems.

In this review, *in situ* remediation refers to treatment strategies in which PFAS capture and degradation occur directly within the contaminated aquifer or subsurface environment, without extraction of contaminated groundwater for external treatment. The term further includes integrated systems where capture and degradation occur locally within the same material system or treatment unit. This definition emphasizes direct subsurface treatment and functional colocalization of remediation processes. Synergistic methods are defined here as integrated systems in which adsorption and degradation processes are mechanistically coupled, such that adsorption actively enhances catalytic transformation. This synergy may arise from strong affinity between PFAS and the support material, interfacial preconcentration of target molecules at reactive sites, improved mass transfer, or stabilization and regeneration of catalytic functionality. Under this definition, synergy implies performance that cannot be achieved by adsorption or degradation alone, nor by their simple sequential combination.

This review integrates and critically evaluates recent advances in PFAS remediation, with particular emphasis on synergistic strategies in which PFAS capture and degradation are functionally coupled. With this framework, special attention is given to biobased adsorbents and photocatalytic materials, which represent promising platforms for the coupled sequestration and destruction of PFAS. Beyond material design and treatment performance, the review explores the roles of life cycle assessment (LCA) and artificial intelligence/machine learning (AI/ML) in evaluating, optimizing and guiding PFAS remediation strategies. Such approaches have the potential to overcome the limitations of conventional *ex situ* methods^51,52^ while supporting the development of sustainable *in situ* remediation systems, which provide safer, more adaptive, and environmentally friendly alternatives. By highlighting these innovations, we aim to support the development of PFAS remediation strategies that are both effective and aligned with global sustainability goals.

## 2 Methods for PFAS Mitigation

### 2.1 “Capture” – Adsorption of PFAS

#### 2.1.1 Adsorption Mechanism

PFAS behave as polar molecules with anionic charges at the functionalized end, and these groups include sulfonic groups or carboxylic acid groups.^53^ This polarity leads to high solubility in a polar solvent, such as water or methanol. There is also a strong electronegativity due to the fluorine atoms, displaying the hydrophobic effect over the charged effect.^54^ Thus, a substrate surface with positively charged (protonated) functional groups favors PFAS adsorption and corresponding removal.^55^

The typical PFAS adsorption on adsorbent is proposed to have three stages: (i) external diffusion from bulk solution to the surface of adsorbent, (ii) intraparticle diffusion into the adsorbent pore, and (iii) instantaneous surface adsorption at the active sites.^56^

During the adsorption of PFAS, several molecular interactions, including physical and chemical adsorption, are involved such as electrostatic and hydrophobic interactions.^57^ Physical adsorption typically occurs through Van Der Waals forces or electrostatic attractions between the PFAS molecules in solution (adsorbate) and the functional groups present on the surface of the adsorbent. Physisorption commonly occurs as a multilayer process, takes place rapidly, and requires low activation energy due to the weak interaction energy between the adsorbate and the adsorbent.^58^ Due to the unique molecular properties of PFAS, physical adsorptions that include electrostatic interaction, hydrophobic effects, and self-aggregation (formation of micelles or hemimicelles) have been reported as the main possible mechanisms of adsorption (Figure 1).

**Figure 1:**
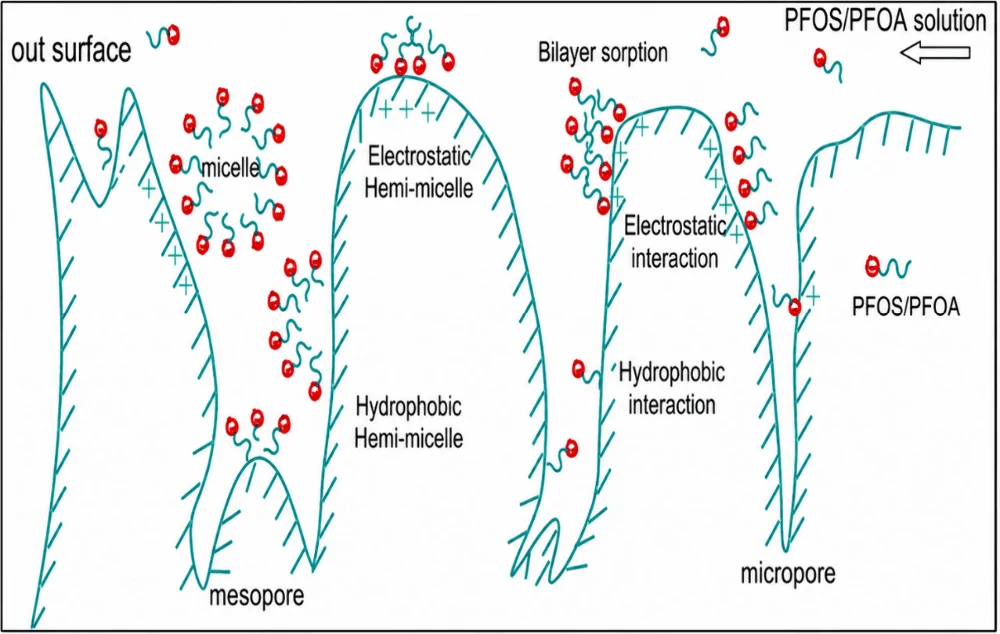
Schematic diagram for the adsorption of PFOS and PFOA on activated carbon fibers. Reused from ref.^59^ Copyright 2017 the Royal Society of Chemistry.

Electrostatic interactions stand for the attraction between the positively charged functional groups of adsorbents surfaces and the anionic functional groups of PFAS. Usually, the adsorption decreased with increasing solution pH, and high adsorption capacity was obtained at the lower pH range.^60^ For example, for activated carbon (AC), decreasing the solution pH from 7 to 3, increased the equilibrium adsorption capacity of PFOA from 174 to 327 mg g^–1^ (0.42 to 0.79 mmol g^–1^) and PFOS from 300 to 345 mg g^–1^ (0.60 to 0.69 mmol g^–1^). It was also reported that some polar functional groups (dipoles), such as amine and hydroxyl groups, display a weak ion-dipole interaction with the anionic PFAS molecules.^61^

For electrostatic interactions, solution pH and ionic strength are important factors dominating the protonation and deprotonation of functional groups. At the same time, the electrical double layer thickness of the adsorbent surface varies depending on the solution pH and ionic strength. Acidic condition is generally more beneficial for PFAS adsorption and the increased adsorption capacity from 900 mg g^–1^ (1.8 mmol g^–1^) at pH 7 to 2750 mg g^–1^ (5.5 mmol g^–1^) at pH 3 of perfluorooctanesulfonate (PFOS) onto AC.^62^ Another example is chitosan, and the primary amine on the glucosamine residues can be protonated over a wide range of pH, behaving in an aqueous solution as a cationic polyelectrolyte. The protonation constants (log K) of chitosan in an aqueous solution vary from 6.3 to 7.3.^63^ At pH 2–4, the chitosan surface is strongly positively charged, which is more favorable for PFAS adsorption.

It was found that, PFAS adsorption capacity was dependent upon the PFAS carbon chain length itself, with higher adsorption for longer chain PFAS.^64^ The reason lies in the hydrophobic nature of long-chain PFAS, which is a result of the electronegativity of the fluorine atoms and the hydrophobic interactions in the adsorption process. Hydrophobic effects refer to the tendency of relatively polar molecules to stick together in aqueous solution, which is commonly observed in surfactant aggregation or lipids assembly.^65^ Another reason is that long-chain PFAS, such as PFOA and PFOS, were able to self-aggregate through the strong hydrophobic interactions of their carbon chain and to form a bilayer structure, or even micelles and hemimicelles.^60,64,66^ Short-chain PFAS, however, are difficult to aggregate resulting in single molecule adsorption on the surface. Self-aggregated structures could be more easily formed when a large number of the PFAS anions interact with the adsorbents with a positively charged surface.^66^

Chemical adsorption, on the other hand forms bonds, such as covalent bonds of the same kind as those operating in the formation of chemical compounds. Chemisorption is limited to a monolayer adsorption, depending on the total surface area.^58^ This process is usually irreversible due to chemical bonding, indicating no recovery via the desorption process. When chemical and physical adsorption takes place simultaneously, any adsorbed layers beyond the first layer are physisorption, making the process fully reversible and dissociative, enabling both the adsorption and desorption processes to be studied. Hydrophobic effects and electrostatic-induced ion-dipole interactions were identified as the factors explaining the adsorption–desorption mechanisms between lignin and PFAS in pulp and paper wastewater systems. The adsorption intensities for the long-chain PFAS were less favorable and consistent with sorption occurring via physisorption, whereas the adsorption intensities of short-chain PFAS were favorable, possibly suggesting chemisorption.^67^

One of the main intermolecular interactions of PFAS with material substrates is hydrogen bonding, which is reported to possess both physical and chemical adsorption properties determined by bond length according to Jeffrey’s classification.^68^ Multiple publications identified hydrogen bonds as the contributors to PFAS adsorption, especially with biomaterials such as chitosan and carrageenan.^69–71^

Adsorption kinetics represents the variation of molecules or chemicals amounts adsorbed to substrate surface as a function of time, and the adsorption rates depend on the collision rate of molecules. The most commonly used kinetic models are pseudo-first-order and pseudo-second-order (Equations S1 and S2) to describe adsorption rates dependence on the concentration of molecules.^72,73^

Adsorption isotherms describe the equilibrium adsorption state of a substance to a solid surface from an aqueous or gas phase under isothermal conditions, when the rate of adsorption is equal to the rate of desorption. These isotherms offer perspectives of the thermodynamic adsorption mechanism, substrate surface properties and the degree of affinity of the adsorbents. The Langmuir model assumes a homogeneous adsorbent surface with energetically equivalent binding sites and monolayer adsorption, which is typically associated with specific interactions such as electrostatic attraction or chemisorption. In contrast, the Freundlich model describes adsorption on heterogeneous surfaces with a distribution of binding energies and is usually associated with multilayer adsorption and nonuniform interactions.

Both Langmuir and Freundlich models (Equations S3 and S4) have been applied for PFAS adsorption, indicating a monolayer adsorption,^69,74–76^ or multilayer adsorption mechanism on the biomaterial surface.^67,77^ The applicability of isotherm models is governed by both adsorbent surface characteristics and PFAS-specific behavior. Materials with relatively uniform and cationic binding sites, such as functionalized polymers and framework-based adsorbents, typically exhibit Langmuir adsorption due to homogeneous surface energetics and monolayer coverage.^69,76^ In contrast, heterogeneous biomaterials, including lignin, polysaccharide, and PEI-modified substrates, tend to follow Freundlich behavior, reflecting a distribution of binding sites and the coexistence of multiple interactions (electrostatic attractions, hydrophobic association, and hydrogen bonding).^67,77^ As a result, the maximum adsorption capacities vary significantly with material structure and functionality, from moderate capacities in the range of 200–300 mg g^–1^ to more than 8000 mg g^–1^, underscoring the critical role of surface chemistry and physics, functional group content, and accessible adsorption sites in governing PFAS adsorption.^67,69,76,77^

The reason for these varying adsorption mechanisms might originate from the properties of biopolymers and processing methods. For example, the adsorption of polyelectrolyte, such as polysaccharides, to pulp cellulosic fibers, demonstrated a pseudo-second-order kinetics and the Langmuir adsorption model. However, following mechanical treatment such as refining, the adsorption mechanisms altered to multilayer Freundlich adsorption due to the increase of surface area and creation of microstructures.^78^ Another reason lies in the monolayer and multilayer formation, which is possibly due to the self-assembly of the PFAS hydrophobic chains.^79^ The PFAS molecules undergo self-aggregation (e.g., hemimicelle or micelle formation) at higher concentrations may lead to deviations from ideal monolayer adsorption and contribute to apparent multilayer behavior. Therefore, isotherm selection should be interpreted as both surface heterogeneity and PFAS aggregation phenomena, rather than solely based on empirical fitting.

Thermodynamic parameters, such as the Gibbs energy change (ΔG), the enthalpy change (ΔH), and the entropy change (ΔS), are calculated from the Vant Hoff’s equation based on the adsorption isotherm constants at equilibrium state (Equation S5). These parameters could further provide physical insights into the thermodynamic properties of the PFAS adsorption (physical or chemical adsorption), and the affinity of molecules to the substrate surface.^80^ The PFOA adsorption has been reported as an exothermic process onto a composite material of a quaternary amine COF combined with chitosan, and the PFOA removal showed a ΔH value more than 40 kJ mol^–1^, suggesting a chemical adsorption of PFOA. The negative ΔS values (−142 J mol^–1^ K^–1^) suggested a decrease in randomness at the solid–liquid interface during adsorption, negative ΔG values at different temperatures indicated a favorable and spontaneous adsorption process.^69^ Similar publication reported the adsorption of PFOA on polyethylenimine modified balsa wood, loofah, and pomelo peel as a spontaneous and exothermic process.^77^

#### 2.1.2 Biopolymer Molecular Interactions with PFAS

Due to the main anionic form of PFAS in aqueous solution, some biopolymers with a natural cationic charge are used as favorable absorbent materials for PFAS, such as chitin and chitosan. The primary amine on the glucosamine residues behaves in an aqueous solution as a cationic polyelectrolyte that attract anionic PFAS head groups via electrostatic interactions. Moreover, the tunable degree of deacetylation in chitosan enables charge-density optimization for different PFAS species, and the polysaccharide backbone enables additional hydrogen bonding. Another significant source of biopolymers are lignocellulosic polymers derived from the pulp and paper industry, including cellulose, lignins, and hemicelluloses. These biopolymers have been widely studied and utilized in the paper, packaging, construction, agriculture, and additive industries, with a recent surge of interest in electrical, energy storage, carbon capture, and food industries.^81–83^ Some detailed structure, chemical formula, and extraction methods are included in Supporting Information (Figure S1). The fundamental analysis of biopolymers interactions with PFAS is still rare and influenced by the biopolymer’s structures and functional groups, PFAS molecules and adsorption conditions such as pH and ionic strength.

The commonly used calculation methodologies to specify the interaction energy, bond length, or binding affinity include density functional theory (DFT) and molecular dynamic (MD) simulations. The DFT calculations and MD simulations were conducted for adsorption of PFOA by a quaternary amine covalent organic framework (COF) with positive surface charge combined with chitosan. The DFT calculations demonstrated that COF exhibited a higher interaction energy with PFOA (33.2 kJ mol^–1^) compared to chitosan with PFOA (23.8 kJ mol^–1^). The adsorption of chitosan depends on pH of the solution, approximately 3–4 for protonation, and the nonprotonated chitosan adsorbs PFOA by hydrophobic interactions and van der Waals forces. The hydrogen bonding and electrostatic interactions also contribute to the adsorption of PFOA by the chitosan chain tethered to COF skeleton.^69^ Another MD simulation was performed for a chitosan/polyethylenimine based composite xerogel adsorption of Perfluorobutanesulfonic acid (PFBS). The PFBS molecule mainly interacted with the amine and hydroxyl functional groups of the composite xerogel, which are also the active sites for hydrogen bonds. The intensity of intermolecular interaction has been computed to be between 18.8 kJ mol^–1^ and 19.6 kJ mol^–1^ (4.5 and 4.7 kcal mol^–1^) with hydrogen bond length <3 Å, suggesting a “strong, mostly covalent” hydrogen bond and high affinity of the xerogel composite with PFBS molecules (Figure 2).^71^

**Figure 2:**
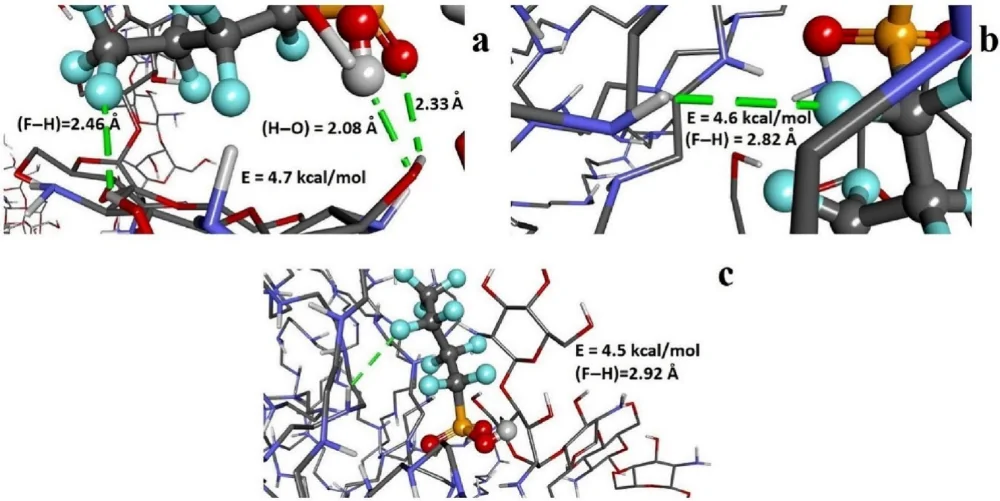
Illustrations of PFBS molecules adsorbed with CS (a), PEI (b), and both CS and PEI (c); nitrogen atoms are in blue, fluorine atoms in light blue, sulfur atoms in yellow, oxygen atoms in red, hydrogen atoms in light gray, and carbon atoms in gray. Reproduced from ref.^71^ Copyright 2023 Elsevier B.V.

Some proteins demonstrated stronger interactions with PFAS than biomaterials, for instance, Mantripragada et al.^84^ prepared an electrospun cellulose (CE) and its derivative cellulose acetate (CA) nanofibrous membranes and further coated these membranes with low-cost soy protein isolate (SPI) for the adsorption of short-chain GenX, obtaining a maximum GenX removal capacity of 363 mg g^–1^ (1.1 mmol g^–1^). The computational analysis for the interaction indicated that SPI has much higher binding affinity with GenX than that of CA or CE while coulomb interaction played much more important role in SPI–GenX interaction than noncoulomb interaction.

#### 2.1.3 Biomaterials Advanced for PFAS Remediation

##### 2.1.3.1 Chitin and Chitosan

Multiple forms of chitin and chitosan materials were investigated for the PFAS adsorption including aerogels, membranes, foams, and beads.^70,71,75,85–87^ Foam, cryogels or aerogels were widely investigated for the PFAS adsorption due to its high total surface area, high adsorption capacity and controllable porosities. García-Castrillo et al.^87^ studied the chitin and chitosan porous cryogels with Fe_3_O_4_ nanoparticles incorporated for PFOA and Perfluorodecanoic acid (PFDA) adsorption (Figure 3). After 50 min of immersion, the adsorption capacities for PFOA and PFDA are 451.4 and 312.3 mg g^–1^, respectively. Sellaoui et al.^85^ reported a polyurethane/chitosan foam for the Perfluorohexanesulfonic acid (PFHxS) and Perfluorobutanesulfonic acid (PFBS) adsorption. The adsorption amount of PFHxS and PFBS was as high as 0.0595 mg g^–1^ and 0.0515 mg g^–1^, respectively, after batch adsorption within Erlenmeyer flasks for 6 h at pH = 4.0. M. R. Shirzad Kebria et al.^71^ reported a chitosan/polyethylenimine xerogel with a maximum PFBS adsorption capacity of 305 mg g^–1^ after 24 h immersion treatment at pH = 6.5.

**Figure 3:**
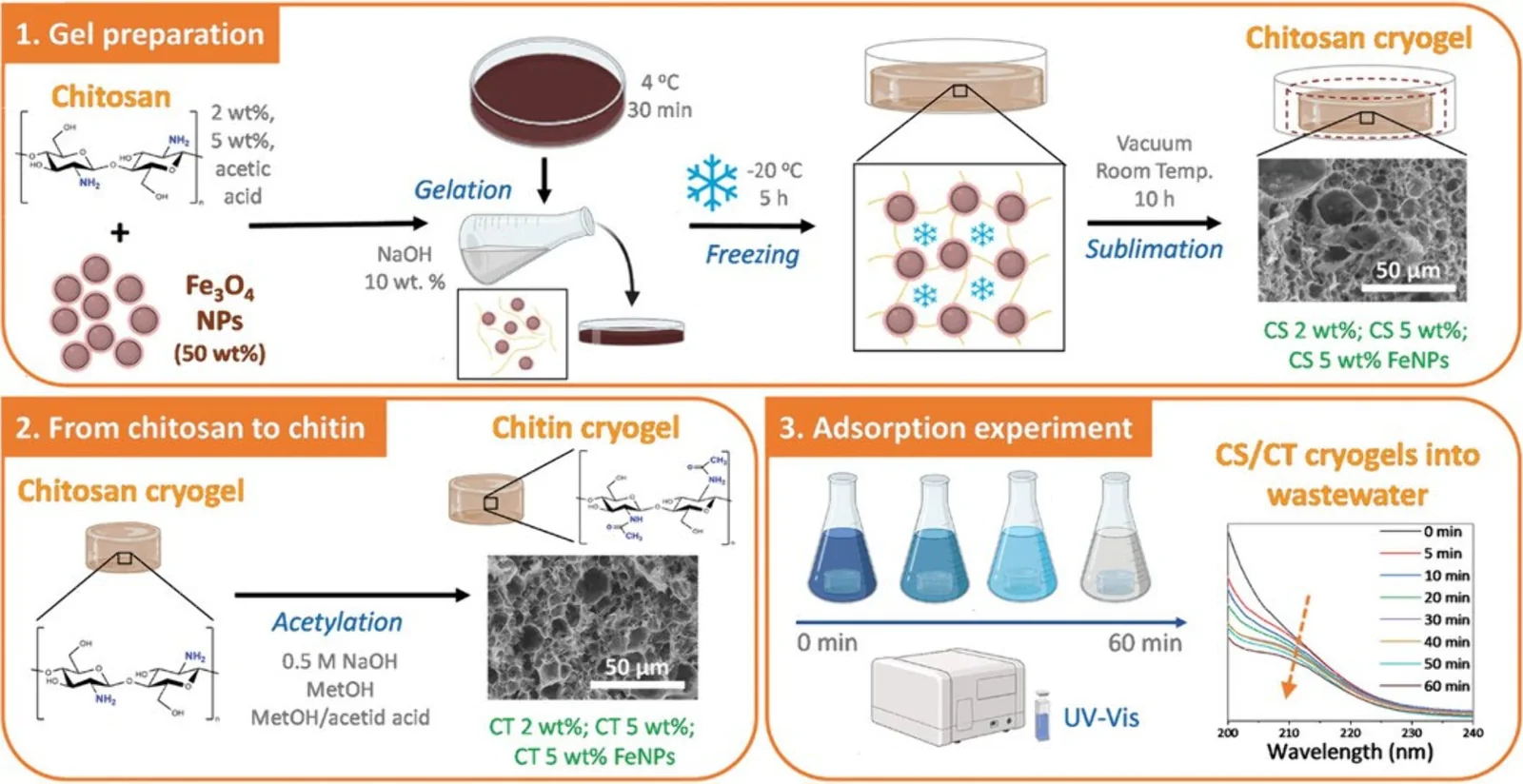
Schematic of the fabrication of magnetic chitosan and chitin cryogels for water purification. Reproduced from ref.^87^ Copyright 2024 Wiley-VCH.

Chitosan composite materials were also investigated for PFAS adsorption. Rahim Shahrokhi and Junboum Park, prepared the crushed chitosan beads (CBs) and polyethylenimine-grafted CBs (GCBs) for a multi-PFAS solution at a concentration of 100 μg L^–1^ for 24 h. For the CBs, the maximum adsorption capacities were reported as 129.4, 207.0, 161.1, and 64.9 mg g^–1^ (312.5, 384.6, 476.2, and 303.1 μmol g^–1^) for PFOA, PFOS, PFBS and Perfluorobutanoic acid (PFBA) respectively. The adsorption values for the GCBs are 230.1, 269.1, 305.8, and 260.2 mg g^–1^ (555.5, 500.0, 1428.6, and 769.2 μmol g^–1^) for the PFOA, PFOS, PFBA, and PFBS, respectively.^75^ Verma et al.^86^ studied cross-linking chitosan and β–cyclodextrin using glutaraldehyde for PFBS adsorption at the maximum capacity of 135.70 ± 25.70 mg g^–1^. The composite material of chitosan with MOFs is a new exploration direction, which combines the advantages of both MOFs pore size control, functional group regulation and chitosan’s positive charge and diverse material formation properties. Zhang et al.^69^ reported a quaternary amine COF with positive surface charge combined with chitosan for the PFOA adsorption at a maximum capacity of 1160 mg g^–1^ (2.8 mmol g^–1^) at pH 5. Another recent work applied a fluoro-functionalized COF (F-COF) with a layer of chitosan on the surface. A high adsorption capacity for PFOS, PFOA, and Ammonium perfluoro (2-methyl-3-oxahexanoic) acid (GenX) reached 8307.1 mg g^–1^, 6177.1 mg g^–1^, and 4603.3 mg g^–1^, respectively. The main contribution factors include the electrostatic interaction, hydrogen bonds, fluorine–fluorine interactions, and channel size selection.^76^

##### 2.1.3.2 Lignins and Modified Lignins

Technical lignins are byproducts of the pulp and paper industries, isolated from black liquor or spent sulfite liquors, including kraft lignin, lignin sulfonates, and soda lignins.^81^ Technical lignins are enriched with hydroxyl groups (OH) including aliphatic OH, carboxylic acid OH, and phenolic OH (C_5_-substituted OH, guaiacyl OH, and *p*-hydroxyphenyl OH).^88,89^ Those hydroxyl groups dissociate at pH = 9 and supply negative charge on the lignin polymers. The carboxylic acid OH groups, on the other hand, dissociate at pH = 6 neutral conditions to supply negative charges.^88^ Thus, it is necessary to have functional group modifications to convert the negative charges into positive ones for the capture of PFAS within the aqueous solutions by the electrostatic forces.

Some typical amination modifications were conducted for this conversion of hydroxyl groups. Li Jinghao et al. reported a lignin modification by the Mannich reaction with formaldehyde solution and polyethylenimine to introduce cationic groups for adsorption (Figure 4).^90^ Another study applied simulated pulp and paper wastewater of four types of PFAS (PFBA, PFBS, PFOA, and PFOS). Kraft lignin adsorption to PFAS with sulfonate functional groups displayed higher affinity, while PFAS with carboxylate head groups remained in the wastewater. The adsorption amount of PFAS increased with PFAS chain length (CF_2_)_n_ among compounds with the same functional group. Long-chain (C ≥ 6) PFAS demonstrated higher sorption compared to short-chain ones (C ≤ 5).^67^

**Figure 4:**
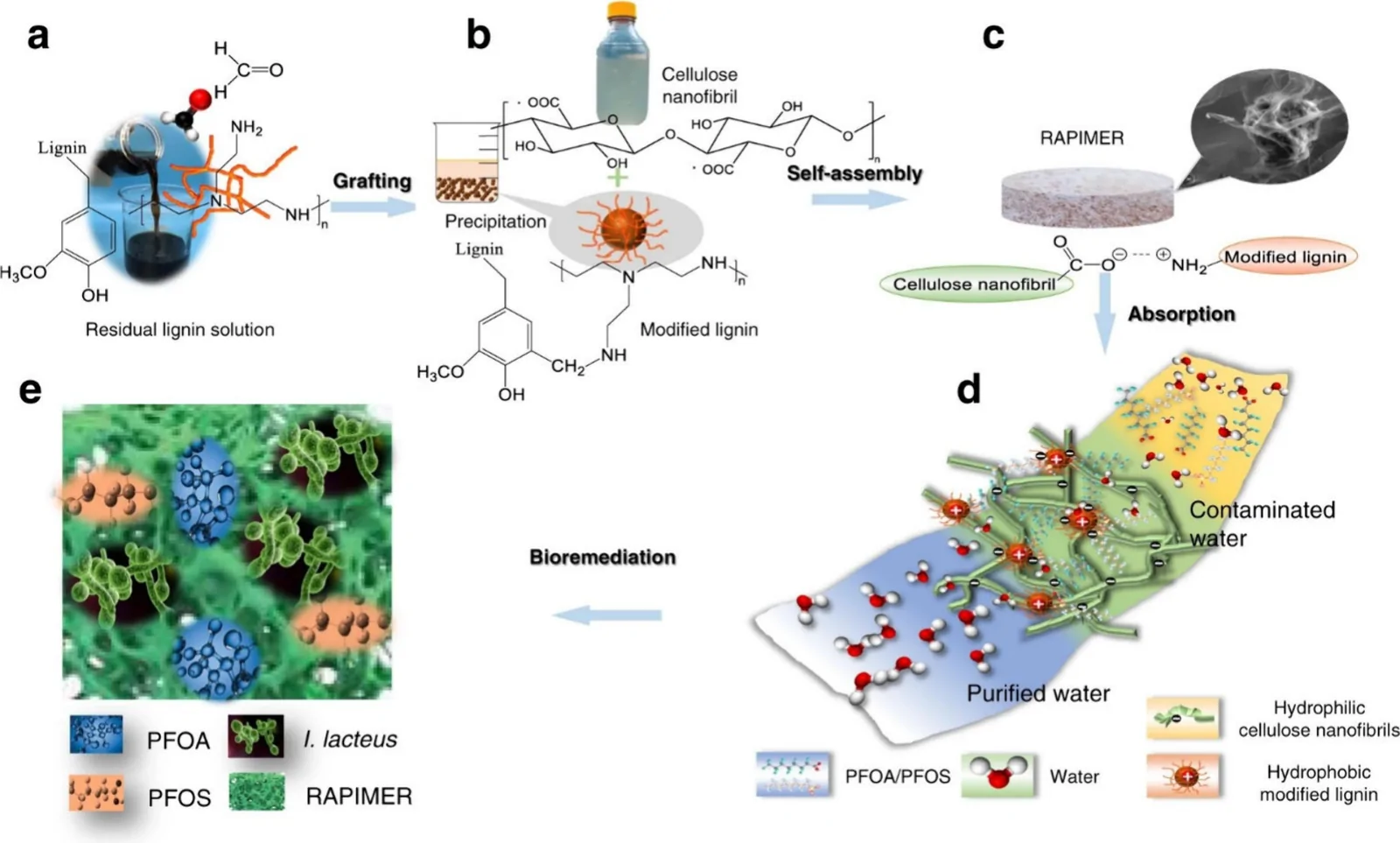
Design illustration, fabrication, adsorption, and fungus degradation scheme of the Renewable Artificial Plant for *in situ* Microbial Environmental Remediation (RAPIMER) system. Reprinted from ref.^90^ under a Creative Commons http://creativecommons.org/licenses/by/4.0/. Copyright 2022 Springer Nature.

##### 2.1.3.3 Cellulose

Varying scales of cellulose were studied for the adsorption of PFAS, from pulp fibers, microfibrillated cellulose (MFC), cellulose nanofibrils (CNF), to cellulose nanocrystals (CNC). The CNC modifications for PFAS adsorption were especially favorable due to the high total surface area, good dispersibility, controlled functional group modifications, and renewable nature. Gomri et al.^91^ reported the homogeneous CNC films from blends of negatively charged CNC and positively charged poly trimethyl aminoethyl methacrylate. The nanocomposite film showed the maximum sorption of 303 mg g^–1^ for PFOA. Zhang et al.^92^ studied the silk fibroin–CNC nanofibrillar and nanoporous membranes for the adsorption of the short-chain PFBA, PFBS, and long-chain PFOA for 24 h. The short-chain PFBA and PFBS showed a higher capacity of 55 ± 21 and 49 ± 17 mg g^–1^, while the long-chain PFOA of 230 ± 57 mg g^–1^.

Some wood pulp fibers or biomass as raw materials were also applied to the PFAS removal and obtained good adsorption capacities. Harris et al.^74^ tested the quaternized wood pulp for PFOS and PFOA adsorption for 24 h achieving 763 mg g^–1^ and 605 mg g^–1^, respectively. Fenghui Qin et al.^77^ utilized polyethylenimine as the modifier for multiple biomass or cellulose-based materials including balsa wood, loofah, and pomelo peel to remove PFOA from water. The maximum adsorption capacity at pH 3.0 was determined to be 279.3, 252.1, and 274.3 mg g^–1^.

##### 2.1.3.4 Other Biomaterials

Another category of biopolymers utilized in recent years is protein for PFAS adsorption. The inherent positive charge, diverse and active in the aqueous matrices offer them advantages for the removal of PFAS. Hernandez et al. developed a protein screening material using proteins including bovine serum albumin (BSA), casein, egg white albumin, lysozyme, and RNase A, for hexafluoropropylene oxide dimer acid (HPFO–DA), PFBA, and PFBS adsorption.^93^ The BSA and lysozyme removed at the maximum of 84–99% of PFOA, HPFO–DA, PFBA, and PFBS at initial concentrations of 0.25 mg L^–1^. Proteins have also been combined with other sustainable adsorbent materials to enhance PFAS removal performance. For instance, biochar, a cost-effective adsorbent with favorable physicochemical properties, has been widely employed for contaminant removal.^57^ Recently, a composite consisting of rice straw-derived biochar and albumin extracted from Moringa oleifera seeds was encapsulated into alginate beads and evaluated for PFAS removal. The PFOS adsorption capacities of albumin-alginate and biochar-alginate beads were reported as 3.17 and 1.57 mg g^–1^, respectively.^79^

The adsorption performance of biomaterials is often sensitive to processing conditions, which can alter their interactions with PFAS molecules. The preparation of biomaterials is also highly dependent on the biopolymer properties, especially the functional group content, accessibility, and protonation behavior, which require further investigation and modification strategies to achieve predictable and reproducible PFAS adsorption. Among these biopolymers, chitin and chitosan remain popular due to their positive surface charge under acidic conditions, enhancing PFAS binding. Additionally, lignin and cellulose, widely available from the pulp and paper industry, have gained increasing interest as sustainable options for PFAS adsorption. In particular, the amphiphilic nature of lignin enables simultaneous interaction with the hydrophobic fluorinated backbone and polar PFAS headgroups, a functional advantage over purely charge-based adsorption systems, with or without further modifications, for wastewater treatment applications. With further amination, chemical doping, and composite formation could effectively introduce cationic groups and porosity, improving adsorption of long-chain and sulfonate PFAS. Future biopolymer-based PFAS adsorbents will develop beyond bulk chemical modification toward controlled fractionation and structure–function design.

While adsorption plays a crucial role in capturing PFAS from contaminated systems, it does not destroy the compounds. Therefore, attention has increasingly shifted toward degradation technologies, such as biodegradation, photodegradation, electrochemical degradation, which aim to break down PFAS molecules up to less harmful byproducts. Given the recent dedicated coverage of biodegradation advances elsewhere,^94^ the following section focuses on photocatalytic degradation, a promising method for the effective destruction of PFAS in environmental matrices.

### 2.2 “Degrade” – Photoinduced Degradation of PFAS

#### 2.2.1 Photolysis of PFAS

Direct photolysis of PFAS in aqueous systems typically yields limited degradation efficiency and requires irradiation at very short, highly energetic wavelengths. This requirement entails substantial energy consumption, raises safety concerns, and is further hindered by the strong absorption of high-energy photons by water. Consequently, PFAS photolysis often leads to incomplete transformation, generating persistent reaction intermediates, commonly shorter-chain PFAS, which may retain environmental mobility and recalcitrance. In light of these limitations, and given that this review aims to provide a forward-looking framework for the development of light-mediated *in situ* technologies for PFAS degradation in water, particularly those relying on photocatalytic pathways, the state of the art on PFAS photolysis and its mechanistic aspects is addressed in the Supporting Information (Section 4).

#### 2.2.2 Photocatalytic Degradation of PFAS

Photocatalytic degradation of PFAS can be achieved through both homogeneous and heterogeneous approaches. In homogeneous photocatalysis, all reactants and the photocatalyst are present in the same phase, which often leads to higher reaction rates. However, these systems are susceptible to deactivation due to limited photostability, which restricts their long-term performance.^95,96^ In addition, challenges related to catalyst separation and reuse raise environmental and economic concerns.

In contrast, heterogeneous photocatalysis involves catalysts in a different phase (typically solid) than the aqueous solution, and is generally preferred for practical applications. Within this category, two main classes can be distinguished: heterogeneous organic photocatalysts and semiconductor-based photocatalysts. The heterogenization of organic photoactive molecules has been explored as a strategy to enhance the stability of photosensitizers that perform well under homogeneous conditions. However, despite promising activity, this approach has largely remained at the laboratory scale due to the limited stability of the photosensitizers, which hinders practical implementation.^96^ Nevertheless, recent work has demonstrated highly stable porphyrin aggregates acting as visible-light-driven photocatalysts, capable of achieving near-complete defluorination of PFAS without the need for chemical additives.^97^

Despite these advances, semiconductor-based photocatalysts generally offer greater advantages in terms of stability, reusability, and ease of integration into engineered water treatment systems.^98–100^ These features make them particularly suitable for in situ applications, such as the Compound Parabolic Concentrator (CPC) system developed in the SOLARDETOX project (HIDROCEN, Madrid).^101^ In light of these considerations, this review focuses on semiconductor-based photocatalysts, given their superior stability and suitability for long-term applications.

##### 2.2.2.1 Semiconductor-Based photocatalysis

Semiconductor photocatalysts have been widely studied for PFAS degradation due to their stability, cost-effectiveness, and photoreactivity.^102,103^ The mechanism of semiconductor-based photocatalysis is explained in detail and could be found in the Supporting Information (Section 5).

A wide variety of semiconductor-based photocatalysts have been studied for PFAS degradation (Table S3). Unsurprisingly, titanium dioxide (TiO_2_) stands out for its chemical stability, low cost, nontoxicity, and UV-light responsiveness.^104^ Its high efficiency has been demonstrated even when used as a raw TiO_2_-rich mineral such as ilmenite, achieving 99.7% PFOA degradation and 97.1% defluorination under UV–C (254 nm) irradiation in the presence of NaNO_3_, along with 97.3% PFOS degradation.^47^ However, although the direct use of raw minerals represents an attractive strategy for in situ applications, particularly when naturally available, impurities may promote charge carrier recombination or interfere with process implementation in water treatment systems. Therefore, refined TiO_2_ is generally preferred, where commercial Degussa P25 TiO_2_ stands out as the benchmark photocatalyst due to its well-defined properties and superior photoactivity.^105^ Its high activity has been further demonstrated in the presence of oxalic acid at acidic pH (pH 3), achieving 87% degradation and 16% defluorination of PFOA over 3 h under 254 nm UV irradiation in a nitrogen atmosphere.^106^ Interestingly, its performance dropped under oxygen-rich conditions, meaning that when a hole scavenger is present (oxalic acid), the degradation mechanism is governed by electron transfer (see Equations S11–S16 in the Supporting Information). This observation is particularly relevant under more realistic conditions, where organic compounds can scavenge photogenerated holes, thereby enabling photogenerated electrons to attack C–F bonds via hydrated electrons or CO_2_^•–^ intermediates, facilitating PFAS reduction.^107^

Despite its advantages, TiO_2_ has notable limitations. Its wide bandgap (3.2 eV vs NHE at pH 7) restricts solar light utilization to approximately 4%, while rapid electron–hole (e^–^/h^+^) recombination significantly reduces photocatalytic efficiency.^108,109^ One strategy to address these drawbacks is the combination of TiO_2_ with mixed metal oxides (MMOs), which has been shown to extend light absorption into the visible region. For instance, an MMO–TiO_2_ photocatalyst obtained via thermal treatment of ZnAl–TiO_2_ achieved 96% PFOA degradation within 4 h under visible light at pH 3.^110^ Plasmonic modification of TiO_2_ with Bi nanoparticles enhances light harvesting and charge separation through the localized surface plasmon resonance (LSPR) effect.^111^ This strategy enabled 99.3% PFOA removal and 58.6% defluorination within 30 min under 365 nm irradiation, outperforming several benchmark photocatalysts including TiO_2_, Ga_2_O_3_, Bi_2_O_3_, and In_2_O_3_. Furthermore, complete removal and 59.3% defluorination were achieved under natural sunlight within 2 h.^112^

Recent computational studies using GPAW in combination with the Atomic Simulation Environment have provided further insight into plasmon-assisted PFAS degradation.^113^ These results indicate that photocatalytic efficiency depends strongly on the interaction between PFAS molecules and the plasmonic material (e.g., Ag or Au). Ag nanoclusters promote PFAS degradation via hot-electron transfer, with degradation efficiency depending on both PFAS structure and cluster size.^114^ Although these findings are primarily based on recent simulations and require further experimental validation, they provide valuable insight into the role of plasmonic materials in PFAS degradation. Such understanding may guide the rational design of hybrid plasmonic–semiconductor photocatalysts. Similarly, improving charge separation through heterojunction engineering can significantly enhance PFAS degradation. For example, an S-scheme ReS_2_/TiO_2_ heterostructure achieved 98% PFOA degradation and ∼80% defluorination within 120 min under UV irradiation at pH 3. The improved performance was attributed to an internal electric field (IEF) generated by ReS_2_, which promoted charge separation and electron transfer, thereby enhancing photocatalytic activity.^115^ Recently, the combination of TiO_2_ with carbon-based materials has emerged as an effective strategy to extend light absorption toward longer wavelengths thereby improving the use of solar radiation. Among these materials, carbon quantum dots (CQDs) have attracted particular attention due to their low toxicity, cost-effectiveness, and wide availability.^116,117^ CQD-modified TiO_2_ photocatalysts, derived from biomass and graphitic carbon sources, have demonstrated promising activity for PFAS degradation, achieving degradation rates of 69% under UVC irradiation (100–280 nm) and 50% under visible light (400–700 nm) at pH 5, with corresponding defluorination efficiencies of 34.5% and 32% for PFOA and short-chain perfluorocarboxylic acids (PFCAs).^118^ Beyond pollutant degradation, this approach can be coupled with simultaneous H_2_ production, highlighting the multifunctionality of CQD-based systems. In these composites, CQDs act as electron acceptors and transfer mediators, facilitating charge separation and transport. Notably, their performance shows a strong size-dependence, which governs their photophysical properties and, consequently, photocatalytic efficiency.^119^ A representative example is the OKCQD/TiO_2_ composite, in which CQDs synthesized from olive kernel biomass were integrated with TiO_2_. This material exhibited high efficiency in the degradation of short-chain PFCAs, achieving degradation rates of 90.1% under UVC irradiation and 80.3% under visible light. Interestingly, short-chain PFCAs (particularly PFBA and PFPrA) displayed slightly higher degradation efficiencies than longer-chain analogues such as PFHxA and PFPeA under both irradiation conditions. This behavior may be attributed to diffusion and adsorption dynamics in mixed PFCA systems. Longer-chain compounds are likely subjected to greater steric hindrance and reduced mass transfer, limiting their adsorption on the TiO_2_ surface, whereas the smaller size and higher mobility of short-chain PFCAs facilitate their access to the photocatalyst active sites. In addition, this composite demonstrated effective performance in complex matrices, with a 71.8% degradation of PFOA in river water and 73.0% in textile wastewater, indicating its potential applicability under environmentally relevant *in situ* conditions. Additional representative examples of TiO_2_–carbon composite photocatalysts are provided in the Supporting Information (Section 5).

Despite the promise of doped titanium-based photocatalysts for PFAS degradation,^120^ a major limitation remains their susceptibility to fluoride-induced surface degradation. The formation of hydrofluoric acid during defluorination may corrode the photocatalyst surface and progressively compromise its efficiency.^121^

Zeolites have also shown promising performance for PFAS adsorption and subsequent degradation, making them attractive candidates for *in situ* water treatment due to their low cost and well-established technological background. In these materials, the SiO_2_/Al_2_O_3_ ratio strongly influences both adsorption and photocatalytic activity, with an optimal ratio around 30. This was demonstrated for Fe-exchanged BEA zeolite (Fe-BEA), which achieved nearly complete PFAS removal after 8 h of UVA irradiation (350 nm).^121,122^ Mechanistic studies combining theoretical calculations and spectroscopic analyses showed that PFOA adsorbs onto the acidic sites of Beta(35) through bidentate/bridging coordination, lowering the C–C bond dissociation energy at the carboxyl end and thereby facilitating degradation. In addition, complexation between Beta(35) and PFOA enhanced photocatalytic performance by generating photogenerated charges in close proximity to the adsorbed PFAS molecules, promoting interfacial electron transfer while reducing competing deactivation pathways.

Indium oxide (In_2_O_3_) emerged as a promising alternative to TiO_2_ photocatalyst due to its strong affinity for PFAS molecules and low •OH production.^123^ In this context, topological modifications from conventional commercial nanoparticles to nanospheres and nanorods significantly improved photocatalytic efficiency by increasing the accessible photoactive surface area. Their mesoporous structure also promoted the diffusion of reactants and intermediates, an important factor governing reaction rates. Nevertheless, short-chain PFAS intermediates were still generated during degradation, as reflected by the gradual accumulation of PFPeA over 8 h, indicating a stepwise defluorination pathway involving sequential CF_2_ removal. To further enhance PFAS adsorption, the incorporation of metal–organic frameworks (MOFs) into these structures produced highly hydrophilic materials that improved PFAS uptake and brought the molecules closer to the photocatalytic active sites. Under this strategy, MOF-derived In_2_O_3_ nanospheres and nanorods achieved complete PFOA degradation within 3 h and full mineralization of short-chain intermediates within 6 h.^124^ Beyond morphological control, the introduction of oxygen vacancies into In_2_O_3_ effectively suppressed photogenerated charge recombination by acting as charge-trapping sites. In_2_O_3_ prepared at lower calcination temperatures (300–400 °C) exhibited higher oxygen vacancy densities and surface areas, resulting in complete PFOA removal within 4 h and defluorination rates of 35–39% after 8 h. In contrast, In_2_O_3_ calcined at 600 °C showed substantially lower activity, reaching only 20% defluorination.^125^ In addition, the presence of Fe^3+^ significantly enhanced the performance of the In_2_O_3_-600, increasing defluorination from 20% to 60%, likely by promoting PFOA coordination and facilitating electron transfer. Cocatalyst incorporation has also been explored to further improve oxygen-vacancy-rich In_2_O_3_ systems. MnO_*x*_-modified oxygen-deficient In_2_O_3_ achieved 99.8% PFOA degradation and 92.4% defluorination within 24 h under acidic conditions.^126^ Unlike previously reported hole-dominated degradation pathways, this material appeared to involve a synergistic contribution of •OH and h^+^, although further photophysical studies are still required to clarify the mechanism. Importantly, oxygen vacancies displayed a strong affinity for PFOA molecules and acted as preferential adsorption and reaction sites. The generation of oxygen vacancies increased the number of unsaturated surface atoms, thereby enhancing PFOA adsorption and promoting subsequent photocatalytic reactions.

Toward the implementation of In_2_O_3_ photocatalysts for *in situ* applications, N-doped In_2_O_3_ prepared by interstitial doping with NH_4_NO_3_ achieved nearly complete PFOA degradation and 36.1% defluorination at pH 5 using only 0.1 mg mL^–1^ of catalyst.^127^ Its degradation efficiency across different water matrices followed the trend: ultrapure water (96.7%) > tap water (58.0%) > secondary effluent (40.7%) > Jiuxiang River water (17.3%), demonstrating a certain degree of robustness under environmentally relevant conditions. In the same study, the effects of several environmental parameters on PFOA degradation were evaluated, including pH, sulfate, chloride, H_2_O_2_, catalyst loading, natural organic matter (NOM), and dissolved oxygen. Among these, pH, sulfate, chloride, and H_2_O_2_ showed the strongest inhibitory effects. Optimal degradation (97.6%) and nearly complete defluorination were achieved at pH 2 in the absence of H_2_O_2_, while a secondary optimum at pH 4.2 resulted in 55.2% degradation and 23.6% defluorination.^128^

Among metal oxides, β-Ga_2_O_3_, the most stable polymorph of Ga_2_O_3_, has shown considerable potential for PFAS degradation due to its strong surface affinity for PFAS molecules and its electron-driven reduction pathway.^100,102^ In particular, the Ga_2_O_3_/persulfate (PMS) system under UV irradiation achieved complete PFOA degradation within 90 min at 254 nm and 60 min at 185 nm, with acidic conditions further enhancing the reaction.^102^ These results supported a degradation mechanism dominated by photogenerated electrons rather than •OH or h^+^, in contrast to many previously reported photocatalytic systems (vide supra). The process also remained effective in real wastewater matrices, achieving 75–85% TOC removal. Mechanistic analyses identified SO_4_^•–^, O_2_^•–^, and e^–^ as the main reactive species involved in degradation.

Beyond the degradation mechanism itself, the implementation of these technologies for *in situ* PFAS remediation must also account for the presence of co-contaminants. In this regard, simultaneous degradation of PFOA and 2,4,6-trichlorophenol (2,4,6-TCP) was achieved using a Ga_2_O_3_–Bi_4_O_7_/F-CTF composite (GaBi/CTF), reaching 93.0% PFOA removal and complete elimination of 2,4,6-TCP within 90 min.^129^ The enhanced degradation rates, 2.9- and 2.8-fold higher for PFOA and TCP, respectively, were attributed to the synergistic effects of Bi_4_O_7_ and the fluorinated covalent triazine framework (F-CTF), which improved charge separation and reactive species generation. Interestingly, PFOA degradation proceeded mainly through h^+^/e^–^ mediated oxidation, whereas TCP degradation was dominated by O_2_^•–^ and •OH attack on its phenolic group. These findings further highlight the potential of Bibased materials for PFAS remediation. This was also demonstrated by the poly­(triazine imide)-modified defective Bi_5_O_7_I PTI/BB photocatalyst, which promoted a coupled mechanism involving reductive hydrogenation and oxidative decarboxylation of PFOA. Under 300 W Xe lamp irradiation, the material achieved 94.8% PFOA removal and 88.1% defluorination within 3 h using 0.5 g L^–1^ catalyst at an initial PFOA concentration of 30 mg L^–1^.^130^

Overall, the reported efficiencies of metal oxide photocatalysts for PFOA degradation follow the order Ga_2_O_3_ > TiO_2_ > CeO_2_ > In_2_O_3_ > CdS; with Ga_2_O_3_ achieving complete PFOA degradation within 10 h under acidic conditions.^131^ Mechanistically, Ga_2_O_3_ predominantly relies on photogenerated electrons, whereas TiO_2_, CeO_2_, and In_2_O_3_ mainly operate through hole-mediated oxidation pathways. These advances demonstrate how the rational design of photocatalytic materials through morphology control, heterojunction engineering, defect generation, and cocatalyst incorporation is progressively defining the roadmap toward robust and efficient materials for the *in situ* degradation of PFAS under environmentally relevant conditions.

#### 2.2.3 A Step toward *In Situ* Photodegradation of PFAS

*In situ* photocatalysis has gained considerable attention in water treatment due to its simplicity, environmental sustainability, and low energy demand. This approach shows promise for the degradation of persistent organic pollutants, heavy metals removal, and microbial inactivation, significantly improving water quality. However, despite its potential, most studies remain at the laboratory scale. Pilot-scale trials are uncommon, and real-world applications in natural water systems are still in early development. Yet, only a few studies have advanced to pilot or demonstration stages, with none reaching commercialization.^132,133^ This gap raises concerns about the feasibility of photocatalysis under real-world conditions. Laboratory experiments typically involve model contaminants in simplified systems such as distilled water, extreme pH levels, or tightly controlled ionic strengths; these do not reflect the complexity of actual wastewater, which contains various impurities, organic matter, and competing reactive species.

Real wastewater systems also pose a major challenge due to PFAS concentrations that are much lower than those used in laboratory experiments. While lab studies often test at milligram-per-liter levels, PFAS in real influent wastewater typically occur in the ng L^–1^ to low μg L^–1^ range. For example, influent levels in Europe range from parts per trillion to parts per billion,^134,135^ and in the U.S., the national mean from 2013 to 2020 was 8.4 ± 0.4 ng L^–1^.^136^ These low concentrations reduce mass transfer efficiency to the photocatalyst surface, a critical limitation in photocatalytic systems. In complex water matrices, reactive species (e^–^, h^+^, radicals) are more likely to react with other organic compounds, reducing their availability for PFAS degradation.^137^ As a result, treatment times may increase, or higher catalyst doses may be required, both of which raise operational costs.

Photocatalyst stability and reusability further complicate implementation. In real water, impurities such as oils, dust, and photoproducts can accumulate on catalyst surfaces, blocking active sites and leading to deactivation.^138,139^ These fouling effects reduce performance and may necessitate frequent regeneration or replacement. Moreover, matrix components can influence PFAS adsorption, alter the energy required to generate reactive species, and impact degradation kinetics, ultimately affecting treatment efficiency and cost-effectiveness.

Engineering challenges, especially related to reactor design, also play a key role. Optimizing light absorption and enhancing mass transfer are critical for efficient *in situ* degradation. Parameters like apparent quantum yield (AQY) and external quantum efficiency (EQE) must be considered to ensure effective photon utilization.^140,141^ However, in low-PFAS-concentration systems, mass transfer is limited under laminar flow. While turbulent flow improves mixing, it introduces air bubbles that scatter light, reducing photon efficiency.^142^ Additionally, photocatalyst aggregation increases particle size and affects distribution, while uneven temperatures and variable water quality can further reduce reactor performance.^142^ Together, these factors contribute to reduced efficiency and higher operational and maintenance costs.

## 3 Synergistic Methods for PFAS Remediation

As a result of the incomplete adsorption and partial mineralization of PFAS, there is an increasing focus on integrating treatment technologies to ensure complete PFAS adsorption and mineralization, in line with existing regulatory standards. Recent advancements have shown that combining various treatment methods can significantly enhance the overall effectiveness of PFAS removal and degradation.^143^ In the context of PFAS remediation, true synergy arises when sorption and catalytic degradation are enabled by a shared material platform, such that PFAS affinity and interfacial accumulation directly promote catalytic efficiency rather than serving merely as a preliminary capture step. While research in this field is still developing, several emerging strategies show strong potential for achieving full PFAS remediation. It is important to distinguish such functionally coupled systems from conventional treatment trains, in which adsorption and degradation are implemented as independent or sequential unit operations. While sequential configurations may be effective, they do not exploit interfacial coupling or affinity-driven enhancement of catalytic reactions, and are therefore considered additive or coupled rather than “synergistic” under the framework adopted in this review. In this section, we concentrate on the combination of adsorption and photodegradation as a primary example of synergistic PFAS remediation (Figure 5). Additionally, we will explore other notable combined treatment strategies that provide important performance benchmarks and inform the development of future synergistic PFAS remediation systems.

**Figure 5:**
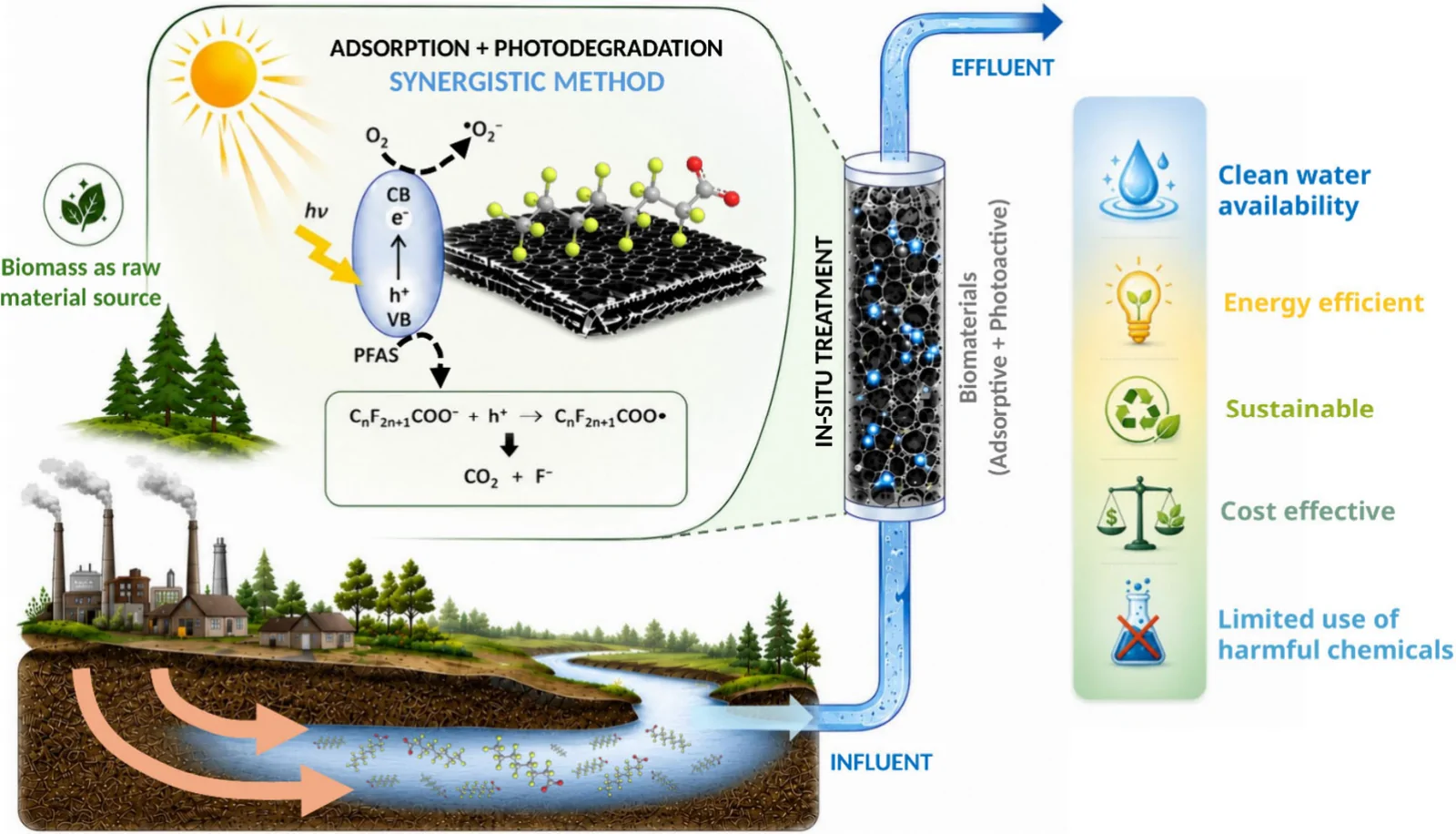
Concept of *in situ* treatment strategy using biomaterials for adsorption and photodriven methods for degradation of PFAS. The figure was conceptualized and drafted by the authors. Selected graphical elements (pipeline, industrial area, water-body cross-section, tree, sun, and material support) were generated using ChatGPT 5.6 (OpenAI) under detailed author-provided instructions.

### 3.1 Adsorption-Coupled Degradation Strategies for PFAS Mitigation

Adsorption effectively localizes and concentrates PFAS, whereas photocatalytic degradation has attracted significant attention for its potential to achieve complete mineralization and defluorination.^49,144,145^ Ren et al.,^146^ combined these approaches using an IXR-photochemical system to enrich and degrade five PFAS (PFPrA, PFHxA, PFOA, PFOS, and GenX), achieving 97.6–100% degradation and 46.6–86.1% defluorination at 1.0 × 10^5^ ng L^–1^.

A similar photocatalytic system was applied to degrade PFOA, PFOS, PFBA, PFBS at 1.0 × 10^4^ ng L^–1^, employing ACs synthesized from biochar, ZVI and chitosan.^147^ Biochar modified with chitosan exhibited complete PFAS removal, and about 86% defluorination. While these studies demonstrate versatility across multiple PFAS molecules, operational complexity remains a limitation. In contrast, the biochar-based photocatalytic system offers improved sustainability and operational simplicity, although large-scale implementation may be constrained by parameters such as pH.

Juve et al.^44^ investigated a “concentrate-and-destroy” strategy combining GAC with In-doped titanium nanotubes (TNT) under photocatalytic conditions. Rapid adsorption (>99% within 30 min) was achieved for PFOA at 1.0 × 10^5^ ng L^–1^, while photocatalyst simultaneously enabled its degradation, confirming that adsorption enhances PFAS-photocatalyst contact and improves mass transfer and degradation efficiency. Similar methods using Fe- and Ga-doped TNTs achieved 66–90% degradation efficiency for PFOA and PFOS within similar timeframes.^148,149^

Despite these advantages, key limitations remain, including the influence of co-contaminants, the need of a comprehensive cost assessment, and reduced effectiveness at low PFAS concentrations. Likewise, Tian et al.^150^ demonstrated the applicability of this approach at a pilot scale, achieving over 90% degradation for several PFASs in landfill leachate. This system represents a significant advance by operating at larger scale with real contaminated water, showing pH insensitivity and high degradation efficiency across diverse PFAS, parameters that support its suitability for *in situ* applications.

Alternatively, iron oxides with carbon spheres, zinc oxide with citric acid-modified graphitic carbon nitride nanocomposites, and carbon-modified bismuth phosphate composites for the removal of PFOA,^151–153^ were also assessed. These materials achieved effective degradation within 4 h and could be reused without chemical regeneration. However, their chemical synthesis raises sustainability concerns, and the time-intensive synthesis process, along with the real-wastewater applicability and cost-effectiveness, still requires thorough evaluation.

#### 3.1.1 Adsorption Coupled with Electrochemical Degradation

Previously studies by Ren et al.^146^ suggested that implementing electrochemical methods can enhance PFAS mineralization. Electrochemical oxidation (EO) offers advantages among oxidation methods, including operation at low temperatures, the absence of harsh chemicals, and minimal waste generation. In contrast, traditional methods like adsorption often require significant energy and chemicals inputs for regeneration.^154^ Therefore, integrating adsorption with EO represents a promising strategy for complete PFAS degradation.

A pilot-scale study by Liang et al.^155^ was conducted for the treatment of ∼ 1,000,000 L PFAS contaminated groundwater from a firefighting site polluted with aqueous film-forming foams (AFFF). This study utilized ion-exchange resins for adsorption and an electrochemical cell with Ti_4_O_7_ electrodes, and approximately, 98% degradation of PFOA and PFOS was achieved. Likewise, Fang et al.^156^ in which PFAS-contaminated ground and tap water were treated using an AIX and a microflow ECO electrochemical oxidation cell (ECO). This method achieved over 99.9% degradation within 24 h and converted more than 85% of organic fluorine into inorganic fluoride. Flow-through columns with resins were especially effective for the removal of PFECAs. Calculations demonstrated that this combined approach, could be more energy-efficient, compared to standalone ECO treatment. However, the process faced challenges, including the use of 80% cosolvent for resin regeneration and inhibited performance in the presence of high dissolved organic matter (DOM). A hybrid approach employing electrocoagulation and electrooxidation was utilized by Shi et al.^157^ to isolate and degrade PFAS. The study further employed acid dissolution to concentrate PFAS from foam residues, followed by degradation using a Ti_4_O_7_ anode. This approach reduced PFAS concentrations in real groundwater systems from ng L^–1^ to mg L^–1^, highlighting its practical applicability.

While many PFAS remediation technologies face limited efficiency, high costs, or excessive sludge, EO stands out for its simplicity, low chemical input, and minimal waste. However, the large-scale application of this method can be a setback due to the concentration effects and limitations of mass transfer.^158^ More research could be conducted on a wide range of PFAS, especially with short-chain PFAS, which are more challenging to remove due to their high mobility, recalcitrant nature, and persistence.^159^

### 3.2 Other Notable Combined Methods

In addition to the methods discussed above, several studies have explored other combined treatment approaches for PFAS remediation, including modified composite materials integrated with photodegradation, adsorption coupled with biodegradation, and membrane filtration combined with advanced oxidation processes, all of which have demonstrated promising results.

Membrane filtration is the most widely accepted approach for PFAS adsorption, which is also already implemented on an industrial scale. Kim et al.^160^ investigated the combination of adsorption and membrane filtration for the removal of PFOA and PFOS at a concentration of 0.01 mg L^–1^. The granular activated carbon and gravity-driven membrane (GAC-GDM) system, a combinatorial approach to remediate PFAS, achieved 97% removal efficiency. Franke et al. (2021),^161^ achieved even higher removal rates (>98%) for 11 types of PFAS and co-contaminants. Two different materials were used for adsorption, which included GAC and strong base anionic resins (AIX) with quaternary amino functional groups. Additionally, a nanofiltration membrane was installed for the treatment of PFAS concentrations ranging from 100–200 ng L^–1^. Furthermore, this study was performed at a pilot scale using actual contaminated water, making it more representative of real-world conditions. Such systems represent highly effective sequential treatment strategies; however, because the individual unit operations function independently, they serve here as comparative benchmarks rather than examples of true functional synergy.

A study by Li et al.^148^ demonstrates how membrane filtration and photodegradation can be used for the degradation of 4 PFCAs, 6 PFSAs, and 2 PFAAs. This pilot-scale study implemented nanofiltration membranes and a photochemical degradation step, achieving approximately 95% degradation. All the PFCAs were degraded in less than 2 h, while PFOA was degraded in less than 4 h. The study also evaluated the effects of chemical sensitizers and sulfite concentrations. Interestingly, energy demands increased with shorter chain PFAS, with the UV reactor and its cooling system being primary energy contributors, but these components could be optimized in full-scale applications. Similarly, Soriano et al.^162^ studied an integrated system of nanofiltration and electrochemical degradation. Starting from 200 mg L^–1^ PFHxA, they achieved 98% degradation within 90 min using real industrial preprocess waters, showing this approach’s practical feasibility.

Lab-scale studies have also shown promising results using composite materials with photodegradation. Hou et al.^163^ fabricated lignin/PVA/Co/Fe MOFs via an electrospinning and *in situ* solvothermal method. Under the influence of solar light, 89.6% of PFOA was degraded, in 3 h, and the materials remained effective over four reuse cycles even with simple rinsing with water. Additionally, a heterogeneous photocatalytic system was developed by Verma et al.^164^ which employed UV–vis/Zn_*x*_Cu_1–*x*_Fe_2_O_4_/oxalic acid, achieving 63 ± 2.7% PFOA degradation at 0.1 mg L^–1^ concentration. The use of solar irradiation, lignin byproducts, simple regeneration, and biomass-derived materials improves both the sustainability and applicability of the aforementioned approaches.

Several studies which use a combined approach that combines adsorption and biodegradation have gained importance in the recent years. As discussed in section 2.1.3.2 Li et al.^90^ introduced RAPIMER, a modified lignocellulosic material that integrates sorption and bioremediation within a single platform. Its amphiphilic cellulose-lignin matrix enables efficient PFAS adsorption while supporting bacterial and fungal growth. White rot fungi can degrade PFAS and the material itself avoiding secondary pollutants, while lignin functionalities promote redox enzyme expression and PFAS decomposition. At a concentration of 100 mg L^–1^ and 25 mg L^–1^ RAPIMER, high adsorption capacities of 3529 mg g^–1^ and 4151 mg g^–1^ were achieved (pH 7.0 ± 0.5) for PFOA and PFOS respectively under theoretical conditions (pseudo-second-order model), and also demonstrated potential in removing co-contaminants such as metals and dyes.

A recent study^165^ used BioSorp Beads (bioactive composite alginate beads embedded with PAC and FeWTR) combined with white rot fungi for sorption and biodegradation of PFAS in stormwater. The use of wastewater byproducts such as ferric sludge, customizability, potential application of this material in conjunction with plant growth, and the complete mineralization of pollutants makes this study much more feasible in real world scenarios.

Hence, the combination of multiple strategies is essential for achieving complete PFAS mineralization and should be prioritized for pilot-scale implementation. The biobased materials, in particular, present a valuable opportunity due to their renewability, low toxicity, and cost-effectiveness. In the next session, we will discuss the optimization of these systems for real-world, large-scale applications through life cycle assessment.

## 4 Life Cycle Assessment (LCA)

In adsorption-based PFAS remediation, LCA provides insights into the sustainability of adsorbent production, operation, regeneration, and disposal, enabling comparison of environmental trade-offs among different treatment strategies. Recent studies have shown that biobased adsorbents derived from lignin, chitosan, and chitin can offer lower environmental impacts than conventional synthetic materials. Several detailed LCA studies have been reported in the literature and representative examples are provided in the Supporting Information (Section 6). Although LCA and techno-economic assessments of *in situ* biopolymer-based remediation technologies are still limited, initial findings indicate significant potential owing to their lower environmental burdens and promising cost-effectiveness.

However, adsorption methods generate concentrated PFAS-laden waste requiring energy-intensive thermal regeneration typically involving drying, desorption, and gasification, thus limiting scalability and increasing costs.^166,167^ Coupled with stricter regulations, there is growing demand for degradation technologies that minimize secondary pollution and allow cost-effective deployment.^168,169^ This has led to increased interest in methods such as ozonation, photocatalysis, electrochemical, thermal, and enzymatic degradation for *in situ* PFAS pollution treatment.^49,94,170,171^ Yet, environmental assessments of these approaches via LCA remain scarce, with few studies addressing GWP, energy use, chemical consumption, or cost. Further examples of these studies are summarized in detail in the Supporting Information (Section 6).

These early LCA studies underscore photocatalysis as a promising strategy for PFAS remediation, particularly given that the solar energy reaching Earth exceeds global energy demand by 3 orders of magnitude. Optimizing photocatalyst efficiency and system design could offer a sustainable path forward to *in situ* application. In addition, recent advances in cost-effective light sources-such as LED technology-and the development of next-generation photocatalysts capable of harnessing solar energy further align this approach with sustainability goals.

### 4.1 Combining Efforts: Environmental Assessment of PFAS Treatment Methods

Although numerous advanced strategies have been proposed for PFAS remediation, many involve severe operational conditions, high energy input, substantial chemical consumption, and reliance on centralized laboratory infrastructure. These factors elevate costs, limit scalability, and reduce the overall practicality and sustainability of such approaches. Researchers have increasingly explored the integration of multiple treatment steps into synergistic remediation methods to enhance PFAS elimination efficiency. However, despite their potential, sustainability assessments of synergetic methods remain uncommon in the literature, which tends to focus more on removal efficiency than on environmental and economic viability.

Some recent studies have addressed this gap. Scotland et al.^172^ investigated electrothermal mineralization of PFOA and PFOS adsorbed on spent GAC via flash Joule heating. This process, using sodium or calcium salts, converts PFAS into inert fluoride salts with over 90% fluorine conversion and >99% PFAS removal. Simultaneously, the GAC is upcycled into flash graphene, offsetting treatment costs by approximately $60–100 kg^–1^. The method operates without solvents or catalysts, and significantly reduces energy demand, greenhouse gas emissions, and secondary waste generation. Techno-economic analysis supports its scalability and cost-effectiveness.

In another study, Li et al.^173^ assessed a sequential treatment train involving ion exchange (IX) followed by electrochemical oxidation (EO). The GWP for 2 orders of magnitude removal of PFOS and PFOA from 1000 m^3^ of water were 72.2 and 84.9 kg CO_2_-eq, respectively, with electricity and resin production identified as major contributors. The study compared several scenarios: (i) single-use IX with incineration (IX + Incin.), (ii) single-use IX followed by EO (IX + EO), and (iii) regenerable IX followed by EO (IX/Re + EO). Results showed that for energy consumption values below 192 kWh m^–3^, IX/Re + EO had the lowest GWP. For values above 695 kWh m^–3^, incineration became more favorable. Between these limits, IX/Re + EO remained the most environmentally efficient. The use of low-carbon energy sources further improves the environmental performance of EO-based scenarios. Similarly, as mentioned before, Li et al.^90^ revealed that RAPIMER was 3–10 times more efficient than activated carbon (AC). The environmental analysis identified the chemical synthesis of cellulose nanofibrils as the most energy-intensive step, whereas electricity consumption contributed only 3.9% to the global warming potential, highlighting the overall sustainability of the process.

Recent reviews have discussed the environmental and economic aspects of PFAS remediation, largely by assessing individual treatment technologies. For instance, Altmeyer Mendes et al.^174^ applied a meta-analysis approach to compare life-cycle impacts and costs across major PFAS remediation strategies, focusing mainly on adsorption- and separation-based technologies, with each technology evaluated independently. Similarly, Song et al.^175^ reviewed the application of LCA to PFAS remediation and showed that such studies remain limited, fragmented, and strongly biased toward physical treatment methods, concluding that LCA for PFAS remediation is still an emerging research area with significant data gaps. Despite these developments, no LCA studies have yet evaluated technologies that couple biopolymer-based adsorbents (e.g., lignin, chitosan) with photocatalytic degradation. In a related study, Liu et al.^133^ reported the energetic analysis of a pilot-scale study where nanofiltration and UV-sulfite treatment were combined for the degradation of PFAS from contaminated groundwater. While a full LCA was not performed, it was demonstrated that this combined method was competitive with other technologies for PFAS destruction. Comprehensive LCAs incorporating both technologies coupled are essential to determine their viability and the deployment of this strategy for PFAS remediation.

## 5 Artificial Intelligence (AI)-Assisted PFAS Remediation

AI, such as machine learning (ML), is a technology that focuses on developing computer algorithms designed to implement knowledge from various fields, such as computer science and information theory, and apply these ideas to mainstream industries. The integration of data-driven approaches improves the data prediction accuracy, reduces experimental consumption, and offers statistical insights into the physical mechanisms. Multiple ML approaches were investigated for PFAS remediation, such as support vector regression (SVR), XGBoost (XGB), random forest (RF), AdaBoost, artificial neural networks (ANN), multilayer perception (MLP), linear regression (LR), and ridge regression (RR).^176^ These algorithms span linear, ensemble, kernel-based, and deep-learning paradigms for PFAS detection, adsorption, membrane separation, degradation, transport, and toxicity prediction.^177–181^ In addition to the broad range of applications, the number of PFAS species involved in the ML models is also high, varying from 1 to 2 types of PFAS, to 43 types of PFAS of varying chain length, head groups, and branching structures.^182–184^ This chemical diversity makes PFAS remediation particularly well suited for ML approaches. Additional detailed examples of ML/AI applications in PFAS remediation strategies are provided in the Supporting Information (Section 7).

AI-driven technologies improve PFAS remediation by capturing complex nonlinear relationships among adsorbent properties, PFAS molecular characteristics, water chemistry, and operational parameters such as pH, temperature, membrane type, and pressure.^183,185,186^ Hybrid models combining response surface methodology (RSM) with ML algorithms such as gradient boosting (GB), RF, and XGB have successfully optimized PFAS adsorption by evaluating factors including pH, pHpzc, surface area, temperature, and PFAS molecular properties, identifying low pH and high pHpzc as favorable conditions for adsorption.^186^ Similar findings were reported using XGB models, which accurately predicted adsorption capacity, equilibrium behavior, and adsorption mechanisms.^187^

Despite these advances, the effectiveness of ML remains strongly dependent on the availability of large and diverse data sets. The complexity of PFAS adsorption interfaces, the diversity of PFAS species, and variations in water matrices limit the amount of available experimental data, restricting mechanistic understanding and predictive accuracy. Current developments in sustainable biomaterials, including cellulose-, lignin-, and chitosan-based adsorbents, present additional challenges due to their multiscale structural heterogeneity. However, AI offers an opportunity to identify the relative importance of factors such as crystallinity, functional group composition, charge distribution, and morphology on PFAS adsorption and degradation.

Furthermore, combined or sequential methods could also play a major role in AI-assisted PFAS remediation. The integration of experimental PFAS adsorption, in situ degradation, and machine learning (ML) modeling can enhance overall system performance and enable more sustainable and energy-efficient design and process optimization.

Future developments are likely to focus on hybrid ML models, that not only accurately predict results, such as adsorption capacity or removal efficiency, with various adsorbents and conditions, but also offer physical insights into the PFAS adsorption and degradation mechanisms. Therefore, the ML approach reduces the need for elongated experimentation, saving time and resources, and exploring the future direction for PFAS remediation in a more sustainable and renewable way.

## 6 Summary and Outlook

This review demonstrates that, despite the growing interest in integrated PFAS treatment strategies, most reported systems still operate as combined or sequential processes rather than as a truly coupled system. Realizing this vision requires moving beyond standalone adsorption or degradation approaches, toward integrated material platforms in which capture and degradation are mechanistically coupled within the same system, thereby enabling effective and sustainable PFAS remediation. Across the reviewed studies, strong PFAS affinity and interfacial preconcentration at reactive sites emerge as key enabling factors that enhance catalytic efficiency, improve removal performance, and reduce secondary waste generation. While adsorption alone is effective for PFAS capture, it does not enable PFAS destruction, underscoring the need for integrated degradation processes. When combined within a single system together with adsorption, photodegradation benefits from PFAS preconcentration, reduced light attenuation, and improved kinetics, supporting the “concentrate-and-destroy” concept. Quantitatively, synergistic adsorption–photocatalysis systems demonstrate near-complete PFAS degradation (∼97–100%), with defluorination degrees reaching up to ∼ 90% at environmentally relevant concentrations (10^4^–10^5^ ng L^–1^) within optimized systems. These systems also exhibit rapid kinetics, achieving >99% removal within 30 min, highlighting the role of PFAS preconcentration in enhancing the rate of mass transfer and catalytic efficiency.

From the available evidence, synergistic degradation-oriented treatment technologies emerge as more promising than isolated methods from a complete mineralization perspective, outperforming adsorption alone (∼50–90% removal without destruction) and standalone photocatalysis (∼30–100% degradation with typically lower defluorination of ∼16–60%). However, their long-term viability depends on sustainable material selection and process design. In this context, biopolymer-based adsorbents offer clear environmental advantages, with adsorption capacities of ∼50–500 mg g^–1^ (up to ∼700–1200 mg g^–1^ in advanced systems) and their integration with photogoverned degradation provides a realistic pathway toward environmentally sound PFAS remediation. By integrating AI with this synergistic strategy, future PFAS remediation strategies can be designed more efficiently and sustainably, especially for complex systems (e.g., multiple PFAS species, heterogeneous interfaces). This review also emphasizes on the importance of LCA of PFAS degradation processes, indicating that complete and sustainable remediation requires both removal and mineralization. Additionally, it also synthesizes selected studies on combined treatment approaches, providing a critical assessment of their advantages and associated shortcomings. Despite these advances, several critical research gaps remain. Mechanistic understanding of adsorption–degradation interfaces is incomplete, particularly for short- and ultrashort-chain PFAS and emerging alternatives, which are increasingly relevant in environmental settings. For adsorption processes, deeper insights are needed into how PFAS interact with biopolymer chemistries, including the contributions of hydrogen bonding, π–π stacking, van der Waals forces, and other hydrophobic interactions. Similarly, for photocatalytic degradation, mineralization pathways remain poorly resolved, especially beyond well-studied model compounds such as PFOA and PFOS. Molecular-level design of tailored adsorbents and photocatalysts, supported by advanced characterization techniques, is essential for bridging the gaps and to accelerate the progress.

Another major limitation lies in the disparity between laboratory conditions and real-world scenarios. Most studies are conducted in simplified aqueous systems at unrealistically high PFAS concentrations, whereas natural waters contain complex ionic backgrounds, dissolved organic matter, co-contaminants, and much lower pollutant levels, which greatly influence the system performance. Future work should prioritize more realistic assessments, including the use of metrics such as total organic carbon (TOC), total fluorine (TF), high-resolution mass spectrometry, and combustion ion chromatography (CIC) to provide accurate measurements of defluorination and mineralization.

Durability and reusability of biopolymer-based adsorbents also remain underexplored. While considered renewable these materials often exhibit limited stability over repeated number of regeneration cycles, particularly under variable pH or in the presence of corrosive agents. Conventional regeneration methods used for AC or synthetic polymers, such as strong solvents or thermal treatments, are not suitable for biopolymers. Addressing these challenges requires the development of hybrid materials that combine biopolymers with biochars, clays, MOFs, or other such materials, with operational structural stability, tunable porosity, and necessary surface modifications, while minimizing secondary waste generation.

Finally, the key challenge lies in scaling up and implementing these methods in practice. Photocatalytic systems, for instance, remain constrained by low energy conversion efficiency and UV-dependence, which restricts their cost-effectiveness at large scales. Reported critical performance metrics such as electrical energy per order (EE/O), external quantum efficiency (EQE), reactor irradiance and catalyst stability is inconsistent, making it difficult to assess real-world applicability. Advances in energy-efficient light sources (e.g., UV-LEDs, solar harvesting technologies) and optimized reactor designs could help bridge the existing gap between laboratory and field scale application.

Looking forward, three transformative directions stand out as promising pathways for PFAS remediation. First, a system design must move beyond proof-of-concept demonstrations toward fully integrated treatment frameworks that deliberately combine technologies, such as adsorption and degradation, into a “synergistic” approach as highlighted in this review. Artificial intelligence (AI) and machine learning (ML) can accelerate this progress by guiding material design, optimizing process parameters, and predicting performance in case-specific applications. Second, sustainability must be central, requiring comprehensive life cycle assessments, cost analyses, and long-term field trials, which are necessary to benchmark the true environmental and economic impacts of these technologies. Finally, alignment with evolving policy and regulatory frameworks is critical, as tightening standards (e.g., EU Drinking Water Directive and US EPA advisories), favor systems that deliver both PFAS mineralization ideally, and sustainable operation which fulfill these regulatory expectations than standalone approaches. In conclusion, PFAS remediation research requires a strategic shift in priorities, from isolated laboratory studies reporting removal efficiencies, toward a holistic system design that addresses mineralization, scalability, sustainability, and real-world applicability.

## Supplementary Material



## Notes

The list of abbreviations; Review methodology; Biopolymers and their intrinsic advantages; Summary of PFAS degradation and defluorination efficiencies under direct photolysis systems; Summary of PFAS degradation and defluorination efficiencies under indirect photolysis systems; Summary of PFAS degradation and defluorination efficiencies under material-assisted heterogeneous photocatalytic systems; Summary of semiconductor-based photocatalysis; Life cycle assessment and AI-assisted PFAS remediation (PDF)
