# Supplementary material for: Synergistic Approaches in PFAS Remediation: Outlook for Sustainable In Situ Mitigation by Combined Adsorption and Photocatalysis
Source: Environ Sci Technol. 2026 Oct 6;60(43):30316–34. doi: 10.1021/acs.est.6c12980 (PMC13738786; doi:10.1021/acs.est.6c12980)
Supplement: Supplementary file 1 [file es6c12980_si_001.pdf]

Synergistic approaches in PFAS remediation: outlook for sustainable *in situ* mitigation by combined adsorption and photocatalysis

Surabhi Sreenitha Raj,[+][a,b] Jingqian Chen,[+][a] Oscar Cabezuelo,[+][a,b] Tetyana M. Budnyak [a,b,c]*

[a] Surabhi Sreenitha Raj, Dr. Jingqian Chen, Dr. Oscar Cabezuelo, Dr. Tetyana M. Budnyak
Department of Earth Sciences, Uppsala University, Villavägen 16, 752 36, Uppsala (Sweden)
E-mail: tetyana.budnyak@geo.uu.se
[b] Surabhi Sreenitha Raj, Dr. Oscar Cabezuelo, Dr. Tetyana M. Budnyak
Wallenberg Initiative Materials Science for Sustainability (WISE), Department of Earth Sciences, Uppsala University, Villavägen 16, 75236, Uppsala (Sweden)
[+] These authors contributed equally to this work

Supplementary Information

Table of contents

1. **The List of Abbreviations**

1.1 Types of PFAS

1.2 Ions/Radicals/Atoms

2. **Review methodology**
3. **Biopolymers and their intrinsic advantages; Adsorption kinetics, isotherms, and thermodynamics**
4. **Photolysis of PFAS molecules**
5. **Semiconductor-based photocatalysis**
6. **Life cycle assessment (LCA)**
7. **Artificial intelligence (AI) assisted PFAS remediation**

List of figures

Figure S1. Schematic representative chemical formula of (A) lignin, reproduced from Meng *et al.*[1] with permission from nature protocols; (B) a cellulose molecule including hydrogen bonding of (1) cellulose **I** and (2) cellulose **II**, reproduced from Heinze, T. and Tashiro, K., & Kobayashi, M. copyright 1991, with permission from Elsevier[2,3] ; (C) α-chitin and β-chitin lignin, reproduced from Pillai *et al.* copyright 2009, with permission from Elsevier,[4] Bai *et al.* copyright 2022, with permission from ACS.[5]

List of tables

Table S1. Summary of PFAS degradation and defluorination efficiencies under direct photolysis systems

Table S2. Summary of PFAS degradation and defluorination efficiencies under indirect photolysis systems

Table S3. Summary of PFAS degradation and defluorination efficiencies under material-assisted heterogeneous photocatalytic systems

Number of pages: 25
Number of figures: 1
Number of tables: 3

1. **The list of Abbreviations**

**Abbreviations**	**Full Form**
AC	Activated Carbons
AI	Artificial Intelligence
ANN	Artificial Neural Networks
AQY	Apparent Quantum Yield
BSA	Bovine Serum Albumin
CB	Conduction Band
CE	Cellulose
CE	Electrospun Cellulose
CNC	Cellulose Nanocrystals
COF	Covalent Organic Framework
CPC	Compound Parabolic Concentrator
CQDS	Carbon Quantum Dots
CS	Chitosan
CT	Chitin
DFT	Density Functional Theory
DOM	Dissolved Organic Matter
EFSA	European Food Safety Authority
EO	Electrochemical Oxidation
EQE	External Quantum Efficiency
GAC	Granular Activated Carbon
GFA	Genetic Function Approximation Algorithm
GWP	Global Warming Potential
ISO	International Organization for Standardization
IXR	Ion Exchange Resins
LCA	Life Cycle Assessment
LR	Linear Regression
LSER	Linear Solvation Energy Relationship
LSPR	Local Surface Plasmon Resonance
MD	Molecular Dynamic
MD	Molecular Dynamic Simulations
ML	Machine Learning
MLP	Multilayer Perception
MLR	Multiple Linear Regression
MMO	Mixed Metal Oxides
Mofs	Metal−Organic Frameworks
NOM	Natural Organic Matter
NS	Nanospheres
PBM	Physics-Based Machine Learning
PEI	Polyethyleneimine
PFBA	Perfluorobutanoic Acid
PTMAEMA	Trimethyl Aminoethyl Methacrylate
PVI	Permutation Variable Importance
REACH	Registration, Evaluation, Authorisation and Restriction of Chemicals
RF	Random Forest
ROS	Reactive Oxygen Species
RR	Ridge Regression
SDGS	Sustainable Development Goals
SPI	Soy Protein Isolate
SVR	Support Vector Regression
TGNCS	Tio_2-Graphene Nanocomposites
VB	Valence Band
WHO	World Health Organization

XGB	Xgboost
NHE	Normal Hydrogen Electrode

1.1 Types of PFAS

6:3-FTCA	6:3 Fluorotelomer Carboxylic Acid
6:3-FTCA	6:3 fluorotelomer carboxylic acid
DIPAP	Polyfluoroalkyl Phosphate Diesters
FHXSA	Fluorohexane Sulfonic Acid
FOSA	Perfluorooctane Sulfonamide
FTCA	Fluorotelomer Carboxylic Acid
FTOHS	Fluorotelomer Alcohols
FTS	Fluorotelomer Sulfonate
FTUCA	Fluorotelomer Unsaturated Carboxylic Acid
GenX	Ammonium perfluoro (2-methyl-3-oxahexanoic) acid
HFPO-DA	Hexafluoropropylene Oxide Dimer Acid
PFAA	Perfluoroalkyl Acids
PFAS	Per- And Polyfluoroalkyl Substances
PFBA	Perfluorobutanoic Acid
PFBS	Perfluorobutanesulfonic
PFCA	Perfluorocarboxylic Acids
PFDA	Perfluorodecanoic Acid
PFDEA	Perfluorodecanoic Acid
PFECAS	Perfluoroether Carboxylic Acids
PFHPA	Perfluoroheptanoic Acid
PFHPS	Perfluoroheptane Sulfonic Acid
PFHXA	Perfluorohexanoic Acid
PFHXS	Perfluorohexane Sulfonic Acid
PFNA	Perfluorononanoic Acid
PFOA	Perfluorooctanoic Acid
PFOS	Perfluorooctane Sulfonic Acid
PFPEA	Perfluoropentanoic Acid
PFPES	Perfluoropentane Sulfonic Acid
PFPRA	Perfluoropropionic Acid
PFSA	Perfluorosulfonic Acids

1.2 Ions/Radicals/Atoms

•OH	Hydroxyl radical
Cn-1f2n+1COO-	Perfluoroalkyl carboxylate
Cn-1f2n-1•	Fluorotelomer alkyl radical
Cn-1f2n-1CH$_2$COO$^-$	Fluorotelomer carboxylate
Cn-1f2n+1COO$^-$	Fluorotelomer unsaturated carboxylate anion
Cnf2n+1•	Perfluoroalkyl radical
Cnf2n+1•	Perfluoroalkyl radical
Cnf2n+1COO-	Perfluorinated carboxylate anion
Cnf2n+1COO$^-$	Perfluorocarboxylic radicals
Cn f2n+1COO•	Perfluoroalkyl carboxylates
Cn f2n+1COO•	Perfluoroalkyl carboxyl radical
$C_nF_{2n+1}COOH$	Perfluorocarboxylic acids
$C_nF_{2n+1}SO_3H$	Perfluorosulfonic acids
Cnf2ncoo•	Perfluoroalkyl carboxyl radical

CO2	Carbon dioxide
$CO_3^{\bullet -}$	Carbonate radical anion
CO42-	Peroxycarbonate
H^+	Photogenerated holes
H2O	Water
HF	Hydrogen fluoride
$K_2S_2O_8$	Potassium persulfate
N2	Nitrogen gas
Na_2SO_3	Sodium sulfite
Nas2o8	Sodium persulfate
O2	Oxygen
$O_2^{\bullet -}$	Superoxide
S2O82-	Persulfate ion
SO4.-	Sulfate radical anion
Ti4O7	Titanium oxide
Znxcu1-xfe2o4	Zinc–copper ferrite

2. Review methodology

This review was conducted using a narrative and critical literature survey focusing on recent advances in PFAS remediation technologies, with particular emphasis on adsorption, degradation, and synergistic treatment. Peer-reviewed articles were identified through database searches in Web of Science, Scopus, and Google Scholar using keywords such as PFAS adsorption, PFAS remediation, biopolymer adsorbents, photocatalytic degradation, in situ treatment, and synergistic remediation. The primary focus was on publications from approximately 2015 to 2025, with the recent 3 years accounting for 47%, the recent 5 years for 64%, and the recent 10 years for 82%. Studies were selected based on relevance to biomaterials, sustainability, material design, mechanistic understanding, and applicability to real or in situ water treatment systems. Some traditional materials, such as activated carbon and synthetic polymer adsorption and remediation publications, were included for comparison. All publications were categorized into adsorption, degradation, and synergistic treatment in Excel and compared for trends and principles based on PFAS species, material preparation, performance, and efficiency. Among them, adsorption accounts for 45%, degradation accounts for 42%, and other hybrid methods account for 13%. Both laboratory-scale and emerging pilot-scale studies were considered, while purely ex situ or non-destructive separation approaches were included primarily for comparison.

3. Biopolymers and their intrinsic advantages

To conquer the increasing global warming and the depletion of petroleum resources, biopolymers and biomaterials have been receiving attention in both research and industrial sectors. PFAS remediation responds to this sustainable trend and shifts the perspective to renewable materials that offer high biocompatibility, biodegradation capacities, and a low carbon footprint.

Chitin is a natural polysaccharide extracted from crustaceans, insects, and fungal sources. It is composed of 2-acetamido-2-deoxy-β-D-glucose units linked through α- and β-(1→4) linkages and is enriched with amino groups, which can also be further defibrillated into chitin nanofibers or chitin nanocrystals.[5,6] Chitosan is a linear copolymer of 2-acetoamido-2-deoxy-β-D-glucopyranose and 2-amino-2-deoxy-β-D-glucopyranose (Figure S1). The protonation constants (log K) of chitosan in an aqueous solution vary from 6.3 to 7.3, depending on the degree of deacetylation and the molecular weight of the polymer.[7]

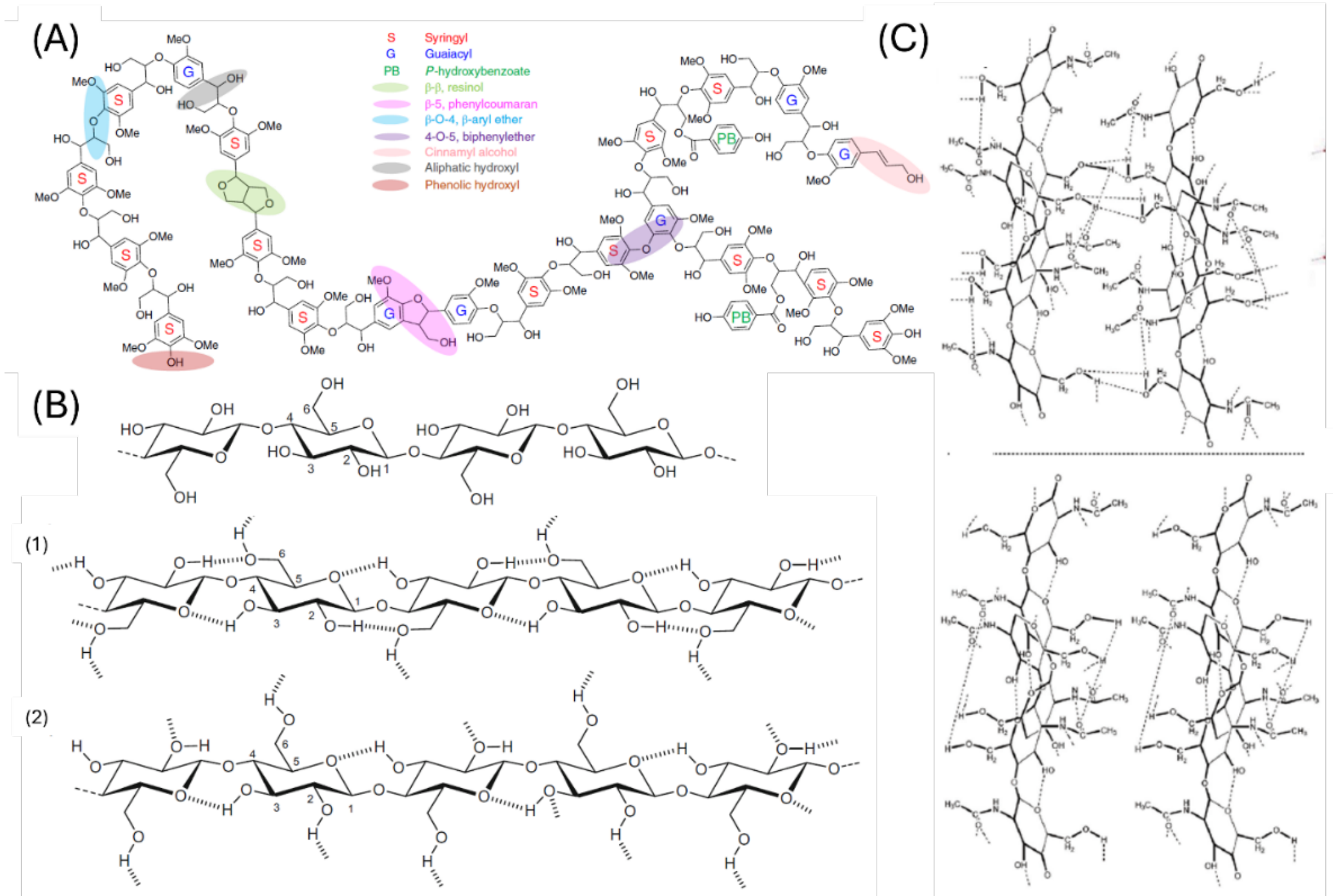

Figure S1. Schematic representative chemical formula of (A) lignin, reproduced from Meng *et al.*[1] with permission from nature protocols; (B) a cellulose molecule including hydrogen bonding of (1) cellulose I and (2) cellulose II, reproduced from Heinze, T. and Tashiro, K., & Kobayashi, M. copyright 1991, with permission from Elsevier[2,3] ; (C) α-chitin and β-chitin lignin, reproduced from Pillai *et al.* copyright 2009, with permission from Elsevier,[4] Bai *et al.* copyright 2022, with permission from ACS.[5]

Cellulose is the most abundant natural biopolymer on earth and is widely studied and applied for biomaterial development. The production of cellulose is mainly from pulp and paper industries, such as kraft pulping and sulfite pulping.[8–10] Cellulose is a polymer of glucose units linked by β-1,4 glycosidic bonds, with the degree of cellulose polymerization ranging from 100 to 20000 anhydro glucose units (Figure 2).[2,3] The crystalline and semi-crystalline structures of cellulose offer high thermal stability.[2] Depending on the application scales, cellulose could be further processed into fibers, fiber fragments, fibrillar fines, micro-fibrillated cellulose (MFC), cellulose nanofibrils (CNF), and cellulose nanocrystals (CNC).[11]

The second most abundant biopolymer extracted from lignocellulosic biomass is lignin. Lignin has an amorphous polyphenol structure characterized by three canonical monolignols: p-coumaryl alcohol, coniferyl alcohol, and sinapyl alcohol, depending on the number of ortho methoxy groups (Figure 2).[12] These monolignols generate the hydroxyphenyl (H), guaiacyl (G), and syringyl (S) subunits, respectively.[13]

Technical lignins are by-products of the pulp and paper industries, isolated from black liquor or spent sulfite liquors, including kraft lignin, lignin sulfonates, and soda lignins.[14] Depending on the processing conditions, the molar mass, sulfur content, and functional group distributions vary widely.[15] Kraft lignin is isolated through hydrothermal treatment of biomass with sodium sulfide (Na_2S) and sodium hydroxide (NaOH), and it is precipitated from black liquor on a commercial scale by the LignoForce, LignoBoost, and WestVaco methods.[8] Consequently, technical lignins generally differ in structure from the native lignin present in biomass due to chemical alterations and cleavage of the β-O-4 bonds during kraft pulping, sulfite pulping, or soda pulping.[8,15]

Different from the technical lignins produced from pulping industries, some lab-scale organo-solvent, hydrolysis, or enzymatic hydrolysis lignins are available for research and development with less alterations of original structures of lignin. Currently, the main usage of lignin precipitated from black liquor is as an energy source for the furnace in pulping mills. Therefore, technical lignins could be regarded as a low-cost, abundant, and commercially available biopolymer for the development of sustainable biomaterials.

Adsorption kinetics, isotherms and thermodynamics

The linearized equations are summarized in Equations S1 and S2. The pseudo-second-order model is the most reported model to describe the PFAS adsorption to biomaterials, which reflects a strong dependence of the adsorption rate constants on PFAS concentration.[16–20]

$$\ln(q_e - q_t) = -kt + lnq_e \quad \text{Equation S1}$$

$$\frac{t}{q_t} = \frac{1}{k(q_e)^2} + \frac{t}{q_e} \quad \text{Equation S2}$$

where k is rate constant (g $mg^{-1}min^{-1}$), q_e is the concentration of molecules (mg g^{-1}) adsorbed at the equilibrium, and q_t is concentration of molecules (mg g^{-1}) adsorbed at any time, t (min).

The Langmuir isotherm was the one of the mostly applied, which was first proposed in 1916 to describe the gas–solid adsorption onto AC.[21] This model assumes identical smooth surface with a finite number of localized sites and ideal monolayer chemisorption of molecules, when there is no lateral interaction or molecule rearrangement.[22] Freundlich model is another widely used two-parameter model, that describes the multi-layer adsorption at the equilibrium state on a non-ideal or heterogeneous surface with adsorption energies having a heterogeneous distribution.[23] The linearized forms of Langmuir and Freundlich isotherms are followed in Equation S3 and S4.

$$\frac{C_e}{q_e} = \frac{1}{K_e Q_{max}} + \frac{C_e}{Q_{max}} \quad \text{Equation S3}$$

$$logq_e = logk + \frac{1}{n} logC_e \quad \text{Equation S4}$$

where C_e is the equilibrium concentration of molecules (mg L^{-1}) in the aqueous phase, S_e is the equilibrium empty site concentration (mg g^{-1}) on the surface of substrate, and Q_{max} (mg g^{-1}) is the maximum adsorption capacity. And k is rate constant (g $mg^{-1}min^{-1}$), q_e is the concentration of molecules (mg g^{-1}) adsorbed at equilibrium state, n is the Freundlich constant.

Enthalpy change (ΔH) is the heat released or absorbed at constant pressure (here, associated with adsorption). The entropy change (ΔS) represents the adsorbate formation at the solid–liquid interface with high randomness ($\Delta S > 0$) or low randomness ($\Delta S < 0$). The Gibbs energy change (ΔG) is applied to determine the degree of spontaneity, and usually a negative value of ΔG indicates a favorable and spontaneous adsorption.[22]

$$lnK_C = \frac{-\Delta H^0}{RT} + \frac{\Delta S^0}{R} \quad \text{Equation S5}$$

where R is the ideal gas constant (8.314 $Jmol^{-1}\ K^{-1}$), T is the absolute temperature (K)

4. Photolysis of PFAS molecules

Although PFAS exhibit weak absorbance at wavelengths commonly used in water treatment (λ < 320 nm), several studies have demonstrated appreciable direct photolysis under specific experimental conditions (Table S1).[24–27]. Hori *et al.*[27] reported 89.5% PFOA degradation after 72 h of UV-vis irradiation (220-460 nm). In a broad study, Xin *et al.*[28] found that FTUCA showed the highest degradation (81%) and defluorination (31%) among 19 representative PFAS under 222 nm light. Also, PFOA removal was achieved in a rate of 25% in a real wastewater matrix within 4 h. Giri *et al.*[29] reported 87.3% degradation and 20.5% defluorination of PFOA under 184 nm light, while combining 185 and 254 nm improved degradation to 100% compared to just 33% under 254 nm. Likewise, Chen *et al.*[30] reported a 61.7% of degradation and 17.1% of defluorination of PFOA under 185 nm irradiation within 2 h. While these findings highlight the potential of direct photolysis under deep-UV exposure, practical limitations remain. Real aqueous environments typically contain low PFAS concentrations than those tested, and natural waters strongly absorb UV light, reducing photon penetration and degradation efficiency.[31] Furthermore, PFAS degradation has been almost negligible under solar radiation.[32,33] Short-chain PFAS are more resistant to photolytic degradation than their long-chain analogues, and the degradation of longer chains can generate further persistent short-chain byproducts, leading to an overall increase in PFAS concentrations in the environment.[34]

Indirect photolysis introduces photosensitizers that can conduct PFAS degradation by both photo-oxidative or photo-reductive pathways. In the photo-oxidative degradation of PFAS molecules, one electron from $C_nF_{2n+1}COO^-$ is abstracted by an oxidant (H_2O_2, CO_3^{-2} or $S_2O_8^{-2}$, among others) to obtain $C_nF_{2n+1}COO\bullet$ (Equation 6) which then suffer the Kolbe-like decarboxylation (Equation S7). Then, a •OH, H_2O can react with the generated $C_nF_{2n+1}\bullet$ radical producing a thermodynamically unstable perfluorinated alcohol (Equation S8) which originate the corresponding acyl fluorides and hydrogen fluoride (Equation S9).[35] Then, the acyl fluorides hydrolyze to the carboxylic acid in the presence of H_2O (Equation S10).[35,36] Finally, the produced $C_{n-1}F_{2n+1}COO^-$ is submitted to a similar degradation pathway to shorter PFCAs molecules. A selection of different studies has been shown in Table S2.

$C_nF_{2n+1}COO^- + \textit{oxidant} \rightarrow C_nF_{2n+1}COO\bullet$	Equation S6
$C_nF_{2n+1}COO\bullet \rightarrow C_nF_{2n+1}\bullet + CO_2$	Equation S7
$C_nF_{2n+1}\bullet + OH\bullet \rightarrow C_nF_{2n+1}OH$	Equation S8
$C_nF_{2n+1}OH \rightarrow C_{n-1}F_{2n-1}C(O)F + F^- + H^+$	Equation S9
$C_{n-1}F_{2n-1}C(O)F + H_2O \rightarrow C_{n-1}F_{2n-1}COO^- + F^- + 2H^+$	Equation S10

Hori *et al.*[27] reported a decrease of PFOA degradation from a 45% achieved from direct photolysis to a 40% in the presence of H_2O_2 due to its UV light absorption below 300nm. The reaction rate constant of hydroxyl radicals with PFOA ($k_{\bullet OH + PFOA}$) is ≤ 10^5 L mol^{-1} s^{-1} , which is much slower compared to the reaction of •OH with most hydrocarbons.[37] In fact, the absence of hydrogen atoms in the PFAS carbon chain, combined with the average C–C bond energy (276.0 $kJmol^{-1}$ vs. 347.0 $kJmol^{-1}$),[38] makes traditional UV/H_2O_2 methods inefficient for PFAS degradation. In comparison, introducing carbonate radicals ($CO_3\bullet^-$), with a redox potential of 1.78 V, to the UV/H_2O_2 system improve its reactivity.[39] Thi, *et al.*[40] demonstrated 100% degradation and 82% defluorination of PFOA with UV/H_2O_2 in the presence of $CO_3^{\bullet-}$. However, high $CO_3^{\bullet-}$ concentrations (>40 mM) can reduce effectiveness by self-quenching process. ($CO_3^{\bullet-} + CO_3^{\bullet-} \rightarrow CO_2 + CO_4^{2-}$).[40]

Persulfate photolysis ($S_2O_8^{2-} + h\nu \rightarrow 2\, SO_4^{\bullet-}$) generates sulfate radicals ($SO_4^{\bullet-}$, E° = 2.5 - 3.1 V)[41] which are highly reactive toward PFAS towards both short- and long-chain PFAS molecules. For instance, Hori *et al.*[42] reported complete PFOA mineralization in 4 hours using 26.8 mM $K_2S_2O_8$ and UV light (254 nm) in the presence of O_2 (0.48 MPa). Chen *et al.*[26] studied the photodegradation of PFOA under 254 nm light and combined 254/182 nm light, both in the presence of $K_2S_2O_8$. Under UV irradiation (254 nm), PFOA degradation and defluorination accounted for 69% and 17%, respectively. In comparison, 90% degradation and 44% defluorination were achieved under 254/182 nm light. Qian *et al.*[43] reported 86% PFOA degradation and 59% defluorination under low-intensity 254 nm light irradiation within 8 hours in the presence of NaS_2O_8, while Wang *et al.*[44] achieved complete degradation (100%) and partial defluorination

(34%) of Perfluorodecanoic acid (PFDeA) under 254/182 nm light irradiation within 6 hours. However, not all PFAS respond equally in the presence of $K_2S_2O_8$: GenX, for example, showed only 5% degradation compared to 26% for PFOA under identical conditions.[45] Similarly, Yang *et al.*[46] demonstrated that PFOS degradation remains limited when persulfate is added, resulting in only a slight improvement in defluorination over 12 hours.

Photoreductive degradation relies on hydrated electrons (e_{aq}^-, E° = -2.9 V), generated from the photolysis of inorganic anions such as sulfite, iodide, and indole compounds, among others.[47] Hydrated electrons can cleave the PFAS head group or facilitate H/F exchange,[48] as systematically reviewed by Liu *et al.*[49] In the H/F exchange pathway, photogenerated electrons play a crucial role in defluorinating α-carbon-fluorine (α-C–F) bonds, influenced by the inductive effect of the carboxylic group. Two sequential H/F exchanges at the α-position yield a fluorinated radical and a $C_nF_{2n}COO•$ intermediate (Equation S11).[50] Although the chain scission mechanism is not yet fully understood, it is proposed that PFOA undergoes α-substitution in the presence of water to form $C_{n-1}F_{2n-1}CH_2COO^-$, which then fragments into $C_{n-1}F_{2n-1}•$ and $C_{n-1}F_{2n-1}COO^-$ species (Equations S12 - S14).[51–53] These intermediates continue to degrade through similar reactions. Alternatively, in the decarboxylative pathway, cleavage of the head group produces a $C_nF_{2n+1}•$ radical (Equation S15), which hydrolyzes to an unstable perfluorinated alcohol (Equation S16). This intermediate eliminates HF and undergoes further hydrolysis, generating $C_{n-1}F_{2n-1}COO^-$, which is then subject to further reductive degradation. However, the exact mechanisms involved in PFAS photoreduction remain unresolved, and the relative contribution of each pathway is still under debate.

$C_nF_{2n+1}COO^- + e_{aq}^- + \rightarrow C_nF_{2n}COO^{•-} + F^-$ Equation S11
$C_nF_{2n}COO^{•-} + H_2O \rightarrow C_nF_{2n}HCOO^- + •OH$ Equation S12
$C_nF_{2n}H_2COO^- + h\nu \rightarrow C_{n-1}F_{2n-1}• + CO_2^{•-} + :CH_2$ Equation S13
$C_{n-1}F_{2n-1}• + CO_2^{•-} \rightarrow C_{n-1}F_{2n-1}COO^-$ Equation S14
$C_nF_{2n+1}COO^- + e_{aq}^- \rightarrow C_nF_{2n+1}• + CO_2^-$ Equation S15
$C_nF_{2n+1}• + H_2O \rightarrow C_nF_{2n+1}OH + H•$ Equation S16

Ren *et al.* [54] investigated the reductive degradation of PFOA in complex water matrices using a UV/sulfite system, and showed high degradation efficiency, strongly influenced by pH and environmental factors. At pH 10, PFOA levels dropped below detection (<10 μg L^{-1}) within 1 hour, achieving 89% defluorination after 24 hours. At pH 12, complete degradation and 98% defluorination occurred within 24 hours, highlighting the role of alkaline conditions in promoting mineralization process. Even at high chloride concentrations (1.709 M, pH 11.25), defluorination reached 97% after 24 hours. The detection of short-chain perfluorinated carboxylic acids (PFCAs, C < 7) and hydrogen-substituted products confirmed H/F exchange and chain-shortening mechanisms. Recently, Lee *et al.*[55] demonstrated degradation of 66.6% PFBS in 6 hours, while PFOS and PFOA were fully degraded in 80 and 20 minutes, respectively. PFOA exhibited the highest defluorination efficiency (77.5%), followed by PFOS (57.8%) and PFBS (42.7%). The authors also support the PFAS degradation pathway via desulfonation, H/F exchange, and chain shortening through direct C−C bond cleavage. Despite the effectiveness of UV/sulfite systems, their overall efficiency is limited by the relatively low quantum yield of e_{aq}^- generation. To overcome this, Liu *et al.*[31] explored PFOA degradation using a vacuum UV/H_2 (185 nm) system in the presence of chloride and sulfate. At pH 12, 90% defluorination was achieved within 80 minutes, and approximately 95% degradation occurred in just 30 minutes. The enhanced performance was attributed to direct UV photolysis of anions. Amador et al.[56] demonstrated that modelling UV/sulfite system for PFAS degradation can be used to optimize the key parameters regarding e_{aq}^- generation, which limits the rates of PFAS degradation during treatment.

Iodide can further improve e_{aq}^- generation and accelerate PFAS degradation.[47,57] O´Connor, *et al.*[58] evaluated a UV/sulfite/iodide system under 254 nm light. For PFOS (20 μM), degradation reached 91% under UV/sulfite and 41% under UV/iodide alone, while the combined system achieved complete mineralization in 45 minutes. PFOA reached >99.9% degradation in 5 minutes with 93% defluorination by 15 minutes. Similarly, Liu, *et al.*[59] reported >99.7% removal of various PFAS (25 μM each) and >90% defluorination in synthetic PFAS-NaCl mixtures using Na_2SO_3 (10 mM) and KI (2 mM) at pH 12 under 254 nm light. However, these systems require alkaline and oxygen-free conditions to avoid e_{aq}^- scavenging.[60] To address this, indole has been proposed as an alternative e_{aq}^- sources. Chen *et al.*[61] achieved 100%

degradation of PFOA (24 μM) and 50% defluorination after 4 h of 254 nm irradiation at pH 6 using 0.4 mM indole. Jin *et al.*[62], developed an indole-based photocatalyst that achieved complete PFOA degradation in 12 h and full defluorination in 4 h; PFOS degradation and defluorination reached 70% under the same conditions. In real water samples (neutral pH, TOC = 8.16 - 28.1 mg L^{-1}), complete PFOA degradation was achieved with 121 μM of indole-nanospheres, and defluorination ranged from 58.9 - 66.5%. Building on this, Wang *et al.*[63] reported complete degradation of PFOA within 4 h under UV with 72% defluorination. PFOS showed 99% degradation and 79% defluorination. Complex PFAS structures such as 6:3 Fluorotelomer Carboxylic Acid (6:3-FTCA) and Hexafluoropropylene Oxide Dimer Acid (HFPO-DA were also fully degraded, with defluorination rates of 66% and 47%, respectively, after 12 h. Indole-based nanospheres create highly reactive zones where reduction (via surface electrons) and oxidation (via hydroxyl radicals) occur simultaneously, even at neutral pH without added buffers, displaying superior performance over conventional e_{aq}^{-} systems.

5. Semiconductor-based photocatalysis

Upon irradiation with light of energy ≥ bandgap, electron-hole (e^- - h^+) pairs are generated: electrons are promoted to the conduction band (CB) while holes remain in the valence band (VB). These charge carriers can migrate to the surface of the semiconductor and induce redox reactions with adsorbed species.[64] Thus, electrons typically reduce acceptors such as O_2, producing reactive oxygen species (ROS) like superoxide ($O_2^{\bullet-}$), while holes oxidize water or organics to form hydroxyl radicals (•OH). However, several studies indicate that •OH have little effect on PFAS degradation,[65–67] as •OH primarily initiates hydrogen abstraction,[68] and excess •OH can compete with PFAS for photogenerated holes, inhibiting their degradation.[69] Hole scavenger experiments confirmed that direct oxidation by h^+ plays a critical role in PFAS degradation.[66,69,70] Here, h^+ induces electron transfer from the carboxylate group of PFAS, forming perfluorocarboxylic radicals ($C_nF_{2n+1}COO^- + h^+ \rightarrow C_nF_{2n+1}COO\bullet$). These radicals undergo stepwise hydrolysis and defluorination, as described in the Supporting Information (Equations S7–S16). Additionally, the alkyl radical ($CnF_{2n+1}\bullet$) can react with oxygen to form a fluorinated peroxoradical (Equation 1),[71] which then combines with another peroxoradical to form oxoradical species (Equation 2). In the presence of photogenerated electrons and water, the oxoradical evolves into an unstable perfluorinated alcohol ($C_nF_{2n+1}OH$, Equation 3), which subsequently hydrolyzes to form carboxylic acids (Equation S9–S10).[36,71] Furthermore, the role of $O_2^{\bullet-}$ in breaking C–F and C–C bonds remain unclear,[70] as previous studies report only a slight decrease in defluorination efficiency (~10% over 24 h) or even negligible effects upon the addition of $O_2^{\bullet-}$ scavengers.[72,73]

$C_nF_{2n+1}\bullet + O_2 \rightarrow C_nF_{2n+1}OO\bullet$ Equation S17

$2C_nF_{2n+1}OO\bullet \rightarrow 2C_nF_{2n+1}O\bullet + O_2$ Equation S18

$C_nF_{2n+1}O\bullet + H_2O + e^- \rightarrow C_nF_{2n+1}OH + OH^-$ Equation S19

Moreover, the combination of TiO_2 with carbon-based materials has provided low-cost and scalable alternatives with demonstrated efficiency for PFAS removal. For instance, a TiO_2/graphene oxide (GO)/polyvinyl alcohol (PVA) nanocomposite achieved removal efficiencies of 95.9% for PFOS and 96.9% for PFOA under 365 nm irradiation at pH 3.[74] GO sheets are especially effective on charge separation due to their high charge mobility and the numerous densities of oxygen-containing functional groups. Thus, GO support acts as an electron acceptor, enhancing the charge separation, and increasing the photocatalytic efficiency. Photogenerated electrons will move toward the GO support and leave behind the holes in the TiO_2 VB. The unpaired π-electrons on GO can interact with TiO_2 to extend the light absorption range leading to effective photochemical activity under visible light irradiation. Moreover, the implementation of the TiO_2/GO/PVA as a nanofilm makes also an easier way to implement it in *in situ* wastewater treatment protocols, benefiting from the already stablished filtration systems which may evolve to introduce light in it. To understand the degradation mechanism, experiments performed in the presence of a quencher revealed

the direct oxidation at the TiO_2 VB as the rate-limiting step, suggesting that strategies aimed at enhancing h^+-driven oxidation can substantially improve photocatalytic efficiency in GO-TiO_2 systems.[75]

Topological modifications and cocatalyst doping of semiconductor photocatalysts can significantly enhance catalytic activity and electron-transfer processes. For example, indium-doped activated carbon-supported titanate nanotubes (In/TNT@AC) achieved >99% PFOA removal within 4 h.[76] The heterojunction structure promoted cooperative adsorption, where the PFOA carboxylate head group interacted with the metal oxide while the hydrophobic tail adsorbed onto activated carbon. This side-on configuration facilitated electron transfer from the carboxyl group to photogenerated holes, while In doping further promoted direct electron transfer and stepwise defluorination. Although these materials are often evaluated under non-realistic conditions, Zhu et al.[77] reported a gallium-doped activated carbon-supported titanate nanotube (Ga/TNT@AC) photocatalyst with demonstrated applicability for *in situ* PFAS treatment under field-water conditions in both batch and continuous-flow systems. In continuous-flow operation, degradation efficiencies reached 99.0%, 88.6%, 73.4%, 66.9%, and 46.2% for PFNS, PFOS, PFPeS, PFHxS, and PFBS, respectively. The addition of 60 μM Fe^{+3} promoted the formation of Fe^{+3}-PFOS and Fe^{+3}-DOM complexes, lowering the activation energy and mitigating dissolved organic matter (DOM) inhibition, while acidic conditions further enhanced degradation.

6. Life cycle assessment (LCA)

García-Castrillo *et al.*[78] evaluated magnetic chitosan and chitin cryogels (max. adsorption: 451 mg g^{-1}). Using ISO 14040/44 standards and Spain's 2023 electricity mix, the GWP for 2 wt% chitosan cryogels was 77.4 kg CO_2-eq kg^{-1}, with chitosan contributing 78% of that impact. Acetic acid and electricity added 9.2% and 9.3%, respectively. Increasing chitosan content to 5 wt% slightly reduced GWP to 67.2 kg CO_2-eq kg^{-1}. Adding Fe_3O_4 nanoparticles (via co-precipitation) brought it down further to 42.6 kg CO_2-eq Kg^{-1}, though the nanoparticles themselves accounted for 18.1 kg CO_2-eq kg^{-1}. Chitin cryogels produced via chitosan acetylation added 75.8 kg CO_2-eq kg^{-1}, driven largely by methanol (76.9%), followed by electricity (12.1%) and sodium hydroxide (5.4%). However, normalizing by output reduced this value to 15.1 kg CO_2-eq kg^{-1}. For comparison, starch aerogels showed a much higher GWP: 3840 kg CO_2-eq kg^{-1} at lab scale, reduced to 1060 kg CO_2-eq kg^{-1} at pilot scale,[79] highlighting the relatively lower environmental impact of chitosan- and chitin-based materials.

Roman, J.B., *et al.*[80] further compared bio-based adsorbents to GAC. While LCA studies for bio-adsorbents remain limited and variable, typical cradle-to-grave GWPs for lignin and cellulosic fibers range from 0.5 to 5 kg CO_2-eq kg^{-1},[81,82] with 2 kg CO_2-eq kg^{-1} being the common value adopted. GAC production, by contrast, incurs 10 - 20 kg CO_2-eq kg^{-1} due to high energy requirements and fossil fuel use.[83,84] Assuming in end-of-life incineration (~70% carbon content), bio-adsorbents' total GWP increases to ~4.6 kg CO_2-eq kg^{-1}.[80] Therefore, to achieve comparable GWP per unit of adsorption, bio-adsorbents would require an adsorption capacity ~30% that of activated carbon. Cost-wise, activated carbon averages €2 Kg^{-1},[85] while the costs of production of bio-based adsorbents are more difficult to define. For instance, the price of lignin ranges from €0.05 to €0.18 kg^{-1},[86] depending on the quality grade. Though LCA and techno-economic evaluations for *in situ* biopolymer use are still emerging, initial findings indicate their potential due to lower environmental burdens and promising cost profiles.

Aratze *et al.*[87] compared ozonation and photo-Fenton processes, finding that ozonation had superior environmental performance due to lower infrastructure and chemical use, though it required more electricity (28% of total impact vs. 8% for photo-Fenton). Photo-Fenton, however, suffers from high CO_2 emissions due to acidification and limited flexibility due to sunlight dependency. A potential improvement lies in conducting the reaction at neutral pH using heterogeneous photocatalysis,[88] which may enhance its environmental assessment. In terms of CO_2 emissions, ozonation produced 0.2 kg CO_2 m^{-3} of treated water, while photo-Fenton emitted 0.86 kg CO_2 m^{-3}. Brüninghoff *et al.*[89] compared electrocatalytic and photocatalytic degradation of 4-ethylphenol in seawater. Despite using a non-optimized photoreactor, photocatalysis achieved high mineralization but maintained high energy demand due to inefficient light

usage. Similarly, Babbit *et al.*[90] showed that photocatalytic oxidation of hazardous air pollutants had lower overall life-cycle impacts (including resource depletion and acidification) than conventional thermal oxidizers with scrubbers.

Solar-driven degradation has also gained attraction. Pesqueira *et al.*[91] ranked solar treatments, with circumneutral photo-Fenton as most intensive (0.3 kg CO_2-eq) and solar photolysis as least (0.025 kg CO_2-eq). Yet, the latter may not provide sufficient toxicity reduction. Solar TiO_2-P25 treatment offered a strong balance, with low impact (0.1 kg CO_2-eq) and reuse over five cycles. Cabezuelo *et al.*[92,93] further reduced environmental burdens by lowering energy demand, material consumption by optimizing TiO_2 loading and applying it in a continuous flow system. This underlines the relevance of process design and operational conditions in reducing the environmental footprint of degradation technologies.

7. Artificial intelligence (AI)-assisted PFAS remediation

One traditional PFAS removal technique is nanofiltration of membranes, and the large quantity of data has supported ML model development in recent years. Permutation variable importance (PVI) analysis revealed that trivalent cations and pH exerted stronger control over PFOS rejection than pressure or PFOS concentration, confirming the central role of electrostatic interactions.[94] Another study also highlights the dominant influence of electrostatic interaction during the PFAS transport across polyamide membranes using the XGB and multimodal transformer models, enabling direct structure-aware learning.[95] Another PVI analysis showed decreasing importance of pressure, initial PFOS concentration, membrane type, trivalent cation, pH, divalent cation, and monovalent cation consecutively, which reveals a clear guidance for the operation conditions.[96] This work represents a transition from empirical regression toward structure-transport-performance relationships.

Several aspects of PFAS remediation technologies are applied with ML techniques, from materials such as AC, resin, biochar, to interaction chemistry, complex water matrices, and degradation performance.[94,95,97–104] For example, 11 PFAS were tested and their corresponding adsorption on varying ACs with parameters of surface area, temperature and pH. The traditional linear solvation energy relationship (LSER) models were used to compare with ML-assisted LSER models, which outperformed traditional LSER models in predicting the adsorption of organic contaminants.[98] Some similar 2D carbon materials were also studied for PFAS adsorption and electrochemical degradation. The integration of physics-based ML models accelerates the optimization, and is more cost-effective for material properties determination.[99] One advanced ML, deep neural networks incorporating noise injection, was applied to model PFNA and PFDA adsorption to biochar from wood and compost, reaching adsorption efficiencies of 90.13% and 85.8%, respectively, which highlighting ML's capacity to manage structural heterogeneity of biomaterials.[100]

Various ML approaches have also been utilized to predict the photocatalytic degradation of PFAS, and ensemble models such as AdaBoost, GBM, and RF overall outperform linear methods. One work reported seven ML algorithms for PFOA photocatalytic degradation, and PVI was used for the relative importance of variables. The AdaBoost, Gradient Boosting Machine (GBM), and RF demonstrated the best performance.[105] Similarly, the GBM algorithm with PaDEL descriptors was demonstrated as a superior technique for the prediction of the relative defluorination ability of PFAS ($R^2 = 0.94$). [104] Some fundamental analysis of the UV-generated hydrated electrons was also considered to play a critical role in the defluorination reaction of PFAS.[104] Both the electrostatic properties and topological structure of the compounds dominated the defluorination ability of the PFAS, suggesting additional electrons on PFAS could cause molecular deconstruction, such as changes in the dihedral angle involved in the carbon chain, the C-F bond and ether C-O bond cleavages. Akber Raza *et al.* (2019) explored an ML approach to predict the C-F bond dissociation energy in PFAS compounds.[106] They utilized algorithms such as RF, LASSO, and feed-forward neural networks to achieve highly accurate predictions. These insights directly inform targeted defluorination strategies rather than indiscriminate destruction.

Table S1. Summary of PFAS degradation and defluorination efficiencies under direct photolysis systems

Targeted PFAS	Experimental conditions	Degradation/Defluorination efficiency	Reaction time (h)	Ref.
**PFOA**	[]$_o$= 1.35 mM; 0.48 MPa of O_2; λ_{em} = 220-460 nm xenon-mercury lamp (200 W)	89.5 % / 95.5%	72	[27]
**PFPrA**	[]$_o$=11-35 μM; pH 8.5; under N_2; λ_{em} = 222 nm (3.14×10^{-6} Einstein L^{-1} s^{-1})	34% /7%	4	[28]
**PFBA**		51% / 30%		
**PFPeA**		57% / 29%		
**PFHxA**		65% / 30%		
**PFHpA**		66% / 30%		
**PFOA**		67% / 24%		
**PFDA**		69% / 27%		
**PFPeS**		< 10% / negligible		
**PFHxS**		< 10% / negligible		
**PFHpS**		< 10% / negligible		
**PFOS**		< 10% / negligible		
**FTS**		negligible / negligible		
**FTCA**		negligible / negligible		
**FTCA**		negligible / negligible		
**FTUCA**		81% / 31%		
**FHxSA**		negligible / negligible		
**FOSA**		negligible / negligible		
**GenX**		30% / 17%		
**diPAP**		negligible / negligible		

**PFOA (nm)**		67% / 24%		
**PFOA (nm)**	$[]_o$=11-35 μM; pH 8.5; under N_2; λ_{em} = 254 nm (2.23 × 10^{-6} Einstein L^{-1} s^{-1})	11% / negligible	5.6	
**PFOA (pH)**	pH 5 to 10.5;	65 – 67% / 24 – 26%	4	
**PFOA (DO)**	Not purged with N_2	67% / 24%		
**PFOA**	Purged with N_2	68% / 26%		
**PFOA (anions)**	$NaHCO_3$, Na_2HPO_4 & NaCl (5000 uM, respectively)	NaHCO3 = 67% /24%<br>Na2HPO4 = 68% / 23%<br>NaCl =74% / 32%		
**PFOA (real water matrix)**	Real wastewater	25% / 7%		
**PFOA (nitrate)**	0 to 36.23 uM	67 to 24% / 24% to negligible		
**PFOA (Ultra pure water)**	$[]_o$ = 2.4 μM; pH 7.7; λ_{em} = 185 nm;	87.3% / 20.5%	3	29
**PFOA (tap water)**	$[]_o$ = 2.4 μM; pH 7.7; λ_{em} = 185 nm;	56.9% / 10.6%		
**PFOA**	$[]_o$ = 2.4 μM; pH 7.7; λ_{em} = 185 nm + 254 nm	100% / 16%	4	107
**PFOA**	$[]_o$ = 2.4 μM; pH 7.7; λ_{em} = 254 nm	33% / 0.7%		
**PFOA**	$[]_o$ = 1 mM; λ_{em} = 185 nm	61.7% / 17.1%	2	30

Table S2. Summary of PFAS degradation and defluorination efficiencies under indirect photolysis system

Targeted PFAS	Experimental conditions	Degradation/Defluorination efficiency	Reaction time (h)	Ref.
**PFOA**	UV/H_2O_2 $[\,]_o$=11-35 μM;<br>$[H_2O_2]$ = 1*106 μM<br>pH 3; λ_{em} = 220-460 nm xenon-mercury lamp (200 W)	40% / 11%	24	27
**PFOA**	$UV/H_2O_2/ CO_3^{\bullet -}$ $[\,]_o$=120 μM;<br>$[H_2O_2]$ = 0.075%<br>$[NaCO_3]$ = 4*104<br>pH 8; λ_{em} = 254 nm (400 W)	100% / 82%	12	40
**PFOA**	UV/ $SO_4^{\bullet -}$ $[\,]_o$=1.35 × 10^3 μM;<br>$[K_2S_2O_8]$ = 6.4 × 10^3 – 5 × 10^4 μM<br>O_2 = 0.48 MPa<br>pH 3; λ_{em} = 254 nm (200 W)	100% / 60%	4	42
**PFOA**	UV/ $SO_4^{\bullet -}$<br>$[\,]_o$= 60 μM;<br>$[K_2S_2O_8]$ = 1.5 × 10^3 μM pH 3; λ_{em} = 254 nm (23 W)	69% / 17%	2	26
	UV/ $SO_4^{\bullet -}$<br>$[\,]_o$= 60 μM;<br>$[K_2S_2O_8]$ = 1.5 × 10^3 μM pH 3; λ_{em} = 254/182 nm (23 W)	90% /44%	2	
**PFOA**	UV/ $SO_4^{\bullet -}$<br>$[\,]_o$= 150 μM;<br>$[NaS_2O_8]$ = 1.5 × 10^3<br>λem = 254 nm (16 W)	86% / 59%	6	43
**PFDeA**	UV/ $SO_4^{\bullet -}$<br>[]o= 100 μM;<br>$[K_2S_2O_8]$ = 1 × 10^3 μM<br>λ_{em} = 254/182 nm (23 W, 62-69 mW*cm^{-2})	100% / 34%	6	44
**GenX & PFOA**	UV/ $SO_4^{\bullet -}$<br>$[\,]_o$= 0.16 mM;<br>$[K_2S_2O_8]$ = 1.6 × 10^2 μM<br>λ_{em} = 254 nm (128 mW*cm^{-2})	GenX<br>5% / 1%<br>PFOA<br>27% / 17%	12	45
**PFOS**	UV/ $SO_4^{\bullet -}$<br>$[\,]_o$= 186 μM;<br>$[K_2S_2O_8]$ = 1.85 × 10^4 μM<br>λ_{em} = 254 nm	Defluorination<br>pH<br>3 / 7 / 11<br>19%/ 17%/ 10%	12	46

**PFOA**	**[]ₒ= 10 mg L-1 (24.15 μM); [Na_2SO_3] = 1 × 10^4 μM pH= 9 to 12 λ_{em} = 254 nm**	**100% (all cases) / 89% (pH9 to 11) ; 98% (pH 12)**	**24**	54
**PFOA, PFOS, PFBS**	$[]_o$= 65.6, 66.1, and 66.2 μM, respectively; [Na_2SO_3] = 2 × 10^4 μM pH= 11 λ_{em} = 254 nm	PFOA, PFOS, PFBS 100%,100%, 66.6%/ 77.5%, 57.8%, 42.7%	6	55
**PFOA, PFOS**	$[]_o$= 2 μM, respectively; [Na_2SO_3] = 1 × 10^4 μM [KI] = 1 × 10^4 pH= 13 λ_{em} = 254 nm	PFOS, PFOA 100%, 100%/ 100%, 93%	6	58
**PFCAs, PFSAs**	$[]_o$= 25 μM, respectively; [Na_2SO_3] = 1 × 10^4 μM [KI] = 2 × 10^3 μM pH= 12 λ_{em} = 254 nm	PFCAs, PFSAs > 99.7%/ > 90%	24	59
**PFOA**	$[]_o$= 24 μM [indole] = 4 × 10^2 μM pH= 6 λ_{em} = 254 nm	100% / 50%		61
**PFOA PFOS**	$[]_o$= 24 μM, respectively [indole-nanospheres] = 121 μM pH= 6 λ_{em} = 254 nm	PFOA, PFOS 100%, 70% / 100%, 68%	12	62
	Industrial wastewater $[PFOA]_o$= 24 μM [indole-nanospheres] = 121 μM pH= 6, λ_{em} = 254 nm	100% / 58.9–66.5%	12	
**PFOA PFOS**	$[]_o$= 24 μM, respectively [indole-nanospheres] = 121 μM pH= 7 λ_{em} = 254 nm	PFOA, PFOS 100%, 99% / 72%, 79%	4	63
**6:3-FTCA HFPO-DA**	$[]_o$= 24 μM, respectively [indole-nanospheres] = 121 μM pH= 7 λ_{em} = 254 nm	6:3-FTCA, HFPO-DA 100% both cases / 66%, 47%	12	

Table S3. Summary of PFAS degradation and defluorination efficiencies under material-assisted heterogeneous photocatalytic systems

Material	Targeted PFAS (mg L^{-1})	Conditions	Degradation efficiency	Test duration (h)	Ref
**TAPP aggregates**	PFOS (0.1)	TAPP aggregates (0.2 g L^{-1}) UV Irradiation (500 W Xenon lamp, 420 nm, 70 mW cm^{-1})	100%	48	108
**Ilmenite mineral sand (raw material containing TiO2)**	PFOA & PFOS (0.2-1.0)	Ilmenite (0.4 g L^{-1}) NaNO3 (10 mM); UV Irradiation (UV-C, 254 nm, 13 W)	PFOA (99.7% - 97.1%) PFOS (99.5% - 97.3%) (pH 6.5)	24	109
**TiO_2 (Degussa, P25)**	PFOA (50)	P25 (0.5 g L^{-1}) Oxalic acid (3 mM) N_2 atmosphere, pH 2.47 (254 nm UV, 23 W low-pressure mercury lamp)	87%	3	35
**Mixed metal oxide-titanium dioxide (MMO-TiO_2) derived from ZnAl-TiO_2 calcination**	PFOA (300)	MMO-TiO_2 (1 g L^{-1}) fluorescent lamps (4 W) as visible-light sources (1.03 mW cm^{-2})	95.5% (pH3)	4	110
**Bi/TiO_2**	PFOA (50)	Bi/TiO_2 ($[]_o$ =0.12 g L^{-1}) Photocatalytic oxidation (365 nm UV, 400 W) & sunlight irradiation (temperature 20–24 °C, light intensity of approximately 900 W m^{-2}) pH 3	UV-(99.3% degradation & 58.6% defluorination) Sunlight- (100% removal rate and 59.3% defluorination)	0.5 h – UV<br>2 h - sunlight	111
**2%ReS_2-TiO_2**	PFOA (2)	25 mg of x% ReS2-TiO_2 photocatalyst in a 0.002 g L^{-1} PFAS solution. Photocatalytic oxidation (365 nm UV) pH 3	Degradation (98%) / Defluorination (75%)	2	112

**TiO_2-CQDs**	PFOA & PFCAs (0.24 μM)	TiO_2-CQDs ([]$_o$ =0.1 g L^{-1}) Photocatalytic oxidation (100-280 nm UVC, 16 W) & Visible (400 – 700 nm, 200 W) pH5	PFOA-UVC (69%)<br>PFOA-Vis (50%)<br>PFHxA-C6-Vis (40%)<br>PFPeA -C5 -Vis (35%)<br>PFBA-C4 -Vis (31.6%)<br>PFPrA-C3-Vis (21.3%)	8	113
**OKCQD/TiO2 composite**	PFOA & PFCAs	OKCQD/TiO2 ([]$_o$ = 1 g L^{-1})<br>100 μg/L PFAS solution (PFHxA (C6), PFPeA (C5), PFBA (C4), and PFPrA (C3) mixture, or PFOA)<br>UVC (4 × 4 W, 254 nm) or visible light (112 LEDs, 200 W, 400–700 nm) pH 5	PFAS mixture<br>90.1 % (UV-C)<br>80.3 % (visible light)<br>PFOA<br>71.8 % (river water)<br>73.0 % (textile wastewater)	8	114
**TiO_2/GO/PVA nanocomposite films**	PFOA & PFOS (0.1)	25 wt% TiO_2/GO; (controlled by 0.01 M NaOH and 0.01 M HCl); Four 15-Watt fluorescent lamps (365 nm)	PFOA (96.9) PFOS (95.9%) (pH 3)	3	74
**TiO_2-graphene nanocomposites (TGNCs)**	PFOA (5)	0.2 g L^{-1}photocatalyst (GO-TiO_2, RGO-TiO_2, GO-P25, or P25) UV-C lamp (Phillips), which had a wavelength of 254 nm with a power of 8 W	GO-TiO2 (93.6%) RGO-TiO2 (99.2%) GO-P25(96%) P25 (16%) pH 4.6 - 4.9	8	75
**In/TNT@AC**	PFOA (0.1)	1 g L^{-1} of In/TNT@AC, UV-C irradiation (254 nm), 25 °C, 1 atm	>99% at pH 7	4	76
**Ga/TNT@AC**	PFNS, PFOS, PFPeS, PFHxS, and PFBS (0.1, each)	1.97 g of column-packed Ga/TNT@AC photocatalyst, UV (254 nm), 60 μM Fe^{+3}	PFNS (99 %), PFOS (89 %), PFPeS (73 %), PFHxS (67 %), and PFBS (46 %) at pH 3.5	24	77
**Fe-BEA**	PFOA (48 μM)	1 g L^{-1} of Fe-BEA; UV-A mercury lamp of 6 W, central wavelength 365 nm	>99% at pH 5	8	115
**BETA(35)**	PFOA (0.05)	0.5 g L^{-1} of BETA(35); 350 W Xe lamp	94.1 at pH 7	48	116
**In_2O_3 nanospheres**	PFOA (10)	In_2O_3 ([]$_o$ =0.2 g L^{-1}) A 32-W UV lamp of 254 nm wavelength (3.5 mW cm−2)	100% (degradation); 100% (mineralization)	3(degradation); 6(mineralization)	117
**In_2O_3-Fe3+**	PFOA (10)	In_2O_3 ([]$_o$ =0.1 - 0.50 g L^{-1}) 254 nm UV light (4.0 mW cm−2)	100%	8	118

**MnOx-Modified In_2O_3**	PFOA (50)	MnO_x-In_2O_3 500 W Xe lamp (100 mW cm^{-1}) for simulated solar light and 500 W Hg lamp for UVA light	99.8% (degradation) pH 1.8 and 3.7 0 and 10% for pH 8.6 and 10.6, respectively	4	119
**N-In_2O_3**	PFOA (0.414)	In_2O_3 ([]$_o$=0.25 g L^{-1}) 350 W xenon (Xe) lamp was used as simulated solar source	87.67%% pH 5	24	120
**In_2O_3 nanoparticles**	PFOA (30)	In_2O_3 nanoparticles (0.1-1 g L^{-1}) low-pressure mercury lamp (15 W) emitting 254 nm UV light	53.50% ± 5.01% (decomposition) at pH = 4.2	3 (degradation) ; 6 (mineralization)	121
**Ga_2O_3**	PFOA concentrations (5 × 10^{-5}, 5 × 10^{-2}, 50)	Ga_2O_3/PMS (0.1 - 2.7 g L^{-1}) and PMS (0.1 to 4.3 g L-1) 32-W low-pressure UV lamp at 254 nm wavelength, 1.71 mW cm2	Ga2O3/PMS (1:1, 2:1, 1:2, 3:1) 33%, 100% and 100%	3	67
**Ga_2O_3-Bi_4O_7 with covalent triazine framework (GaBi/CTF)**	PFOA (1.491 × 10^4) in presence of 2,4,6-TCP 0.072 mol L-1	GaBi/CTF (0.4 g L^{-1}) ultraviolet light (30 W mercury lamp, 254 nm)	100% (pH 3.11); 0% (pH 7)	1.5	122
**PTI/Bi/Bi_5O_7I**	PFOA (30)	PTI/Bi/Bi_5O_7**I** (0.5 g L^{-1}) 300 W Xe lamp (CEL-HXF300) with an AM 1.5 G filter	95% at pH 3	3	123

References

(1) Meng, X.; Crestini, C.; Ben, H.; Hao, N.; Pu, Y.; Ragauskas, A. J.; Argyropoulos, D. S. Determination of Hydroxyl Groups in Biorefinery Resources via Quantitative 31P NMR Spectroscopy. *Nature Protocols* **2019**, *14* (9), 2627–2647.

(2) Tashiro, K.; Kobayashi, M. *Theoretical Evaluation of Three-Dimensional Elastic Constants of Native and Regenerated Celluloses: Role of Hydrogen Bonds. Polymer* **1991**, *32*(8), 1516–1526.

(3) Rojas, O. J. *Cellulose Chemistry and Properties: Fibers, Nanocelluloses and Advanced Materials* **2016**; Vol. 271.

(4) P Pillai, C. K. S.; Paul, W.; Sharma, C. P. Chitin and Chitosan Polymers: Chemistry, Solubility and Fiber Formation. *Progress in Polymer Science* **2009**, *34*(7), 641–678.

(5) Bai, L.; Liu, L.; Esquivel, M.; Tardy, B. L.; Huan, S.; Niu, X.; Liu, S.; Yang, G.; Fan, Y.; Rojas, O. J. Nanochitin: Chemistry, Structure, Assembly, and Applications. *Chemical Reviews* **2022**, *122*(13), 11604–11674.

(6) Kumar, M. N. V. R. *A Review of Chitin and Chitosan Applications* **2000**, *46*(1), 1-27.

(7) Budnyak, T. M.; Vlasova, N. N.; Golovkova, L. P.; Markitan, O.; Baryshnikov, G.; Ågren, H.; Slabon, A. Nucleotide Interaction with a Chitosan Layer on a Silica Surface: Establishing the Mechanism at the Molecular Level. *Langmuir* **2021**, *37* (4), 1511–1520.

(8) Trajano, H. L.; Chen, J.; Yuan, Z.; Zanuso, E. Response of Biomass Species to Hydrothermal Pretreatment. *In Hydrothermal Processing in Biorefineries: Production of Bioethanol and High Added-Value Compounds of Second and Third Generation Biomass* **2017**, 95–140.

(9) Chen, J.; Beatson, R. P.; Tam, K.; Bicho, P.; Trajano, H. L. Kraft Pulping of Softwood Chips with Mild Hot Water-Hydrolysis to Understand the Effects of Wood Chip. *Bioresources* **2022**, *17* (4).

(10) Brown, S. N. M.; Sibellas, A.; Drummond, J.; Chen, J.; Beatson, R. P.; Phillion, A.; Martinez, D. M. Understanding the Origin of Microcompressions in Cellulose Fibres Using 4D X-Ray Micro-Computed Tomography. *Cellulose* **2024**, *31* (3), 1383–1395.

(11) Li, T.; Chen, C.; Brozena, A. H.; Zhu, J. Y.; Xu, L.; Driemeier, C.; Dai, J.; Rojas, O. J.; Isogai, A.; Wågberg, L.; Hu, L. Developing Fibrillated Cellulose as a Sustainable Technological Material. *Nature* **2021**, *590*(7844), 47–56.

(12) Weng, J. K.; Chapple, C. The Origin and Evolution of Lignin Biosynthesis. *New Phytologist* **2010**, *187*(2), 273–285.

(13) Ragauskas, A. J.; Beckham, G. T.; Biddy, M. J.; Chandra, R.; Chen, F.; Davis, M. F.; Davison, B. H.; Dixon, R. A.; Gilna, P.; Keller, M.; Langan, P.; Naskar, A. K.; Saddler, J. N.; Tschaplinski, T. J.; Tuskan, G. A.; Wyman, C. E. Lignin Valorization: Improving Lignin Processing in the Biorefinery. *Science* **2014**, *344*(6185), 1246843.

(14) Barker-Rothschild, D.; Chen, J.; Wan, Z.; Renneckar, S.; Burgert, I.; Ding, Y.; Lu, Y.; Rojas, O. J. Lignin-Based Porous Carbon Adsorbents for CO_2 Capture. *Chemical Society Reviews* **2025**, *54*(2), 623–652.

(15) Eraghi Kazzaz, A.; Fatehi, P. Technical Lignin and Its Potential Modification Routes: A Mini-Review. *Industrial Crops Production* **2020**, *154*, 112732.

(16) He, C.; Yang, Y.; Hou, Y. J.; Luan, T.; Deng, J. Chitosan-Coated Fluoro-Functionalized Covalent Organic Framework as Adsorbent for Efficient Removal of per- and Polyfluoroalkyl Substances from Water. *Separation and Purification Technology* **2022**, *294*, 121195.

(17) Shahrokhi, R.; Park, J. Enhanced Removal of Short- and Long-Chain per- and Poly-Fluoroalkyl Substances from Aqueous Phase Using Crushed Grafted Chitosan Beads: Performance and Mechanisms. *Environmental Pollution* **2024**, *340*, 122836.

(18) Zhang, X.; Wang, S.; Zhu, X.; Zhu, D.; Wang, W.; Wang, B.; Deng, S.; Yu, G. Efficient Removal of per/Polyfluoroalkyl Substances from Water Using Recyclable Chitosan-Coated Covalent Organic Frameworks: Experimental and Theoretical Methods. *Chemosphere* **2024**, *356*, 141942.

(19) Shirzad Kebria, M. R.; Bono, L.; Khoshhal Salestan, S.; Armirotti, A.; Carzino, R.; Athanassiou, A.; Fragouli, D. Efficient Removal of Perfluorobutanesulfonic Acid from Water through a Chitosan/Polyethyleneimine Xerogel. *Chemical Engineering Journal* **2023**, *466*, 143236.

(20) Vakili, M.; Gholami, F.; Zwain, H. M.; Wang, W.; Mojiri, A.; Tomas, M.; Cagnetta, G.; Gholizadeh, R.; Dastyar, W.; Gholami, Z. Removal of GenX by APTES Functionalized Diepoxyoctane Cross-Linked Chitosan Beads. *Journal of Environmental and Chemical Engineering* **2023**, *11*(5), 110539.
(21) Langmuir, I. The Constitution and Fundamental Properties of Solids and Liquids. Part I. Solids. *Journal of American Chemical Society* **1916**, *38* (11), 2221–2295.
(22) Laidler, K. J.; Keith, J. *Chemical Kinetics*; McGraw-Hill: New York, **1965**.
(23) Freundlich, H. Über Die Adsorption in Lösungen. *Zeitschrift für physikalische Chemie* **1907**, 385–470.
(24) Li, J.; Li, B.; Pan, H.; Wei, Y.; Yang, Y.; Xu, N.; Chen, B.; Mohseni, M.; Esfahani, E. B. Total Organic Fluorine (TOF) Analysis by Completely Converting TOF into Fluoride with Vacuum Ultraviolet. *Journal of Hazardous Materials* **2022**, *429*, 128389.
(25) Banayan Esfahani, E.; Mohseni, M. Fluence-Based Photo-Reductive Decomposition of PFAS Using Vacuum UV (VUV) Irradiation: Effects of Key Parameters and Decomposition Mechanism. *Journal of Environmental and Chemical Engineering* **2022**, *10*(1), 107050.
(26) Chen, J.; Zhang, P. Photodegradation of Perfluorooctanoic Acid in Water under Irradiation of 254 Nm and 185 Nm Light by Use of Persulfate. In *Water Science and Technology* **2006**; *54*(11-12), 317–325.
(27) Hori, H.; Hayakawa, E.; Einaga, H.; Kutsuna, S.; Koike, K.; Ibusuki, T.; Kiatagawa, H.; Arakawa, R. Decomposition of Environmentally Persistent Perfluorooctanoic Acid in Water by Photochemical Approaches. *Environmental Science and Technology* **2004**, *38* (22), 6118–6124.
(28) Xin, X.; Kim, J.; Ashley, D. C.; Huang, C. H. Degradation and Defluorination of Per- and Polyfluoroalkyl Substances by Direct Photolysis at 222 Nm. *ACS ES and T Water* **2023**, *3* (8), 2776–2785.
(29) Giri, R. R.; Ozaki, H.; Okada, T.; Taniguchi, S.; Takanami, R. Factors Influencing UV Photodecomposition of Perfluorooctanoic Acid in Water. *Chemical Engineering Journal* **2012**, *180*, 197–203.
(30) CHEN, J.; ZHANG, P.; LIU, J. Photodegradation of Perfluorooctanoic Acid by 185 Nm Vacuum Ultraviolet Light. *Journal of Environmental Sciences* **2007**, *19* (4), 387–390.
(31) Liu, S.; Chen, G.; Shi, Q.; Gan, J.; Jin, B.; Men, Y.; Liu, H. Promotive Effects of Chloride and Sulfate on the Near-Complete Destruction of Perfluorocarboxylates (PFCAs) in Brine via Hydrogen-Tuned 185-Nm UV Photolysis: Mechanisms and Kinetics. *Environmental Science and Technology* **2024**, *58* (23), 10347–10356.
(32) Vaalgamaa, S.; Vähätalo, A. V.; Perkola, N.; Huhtala, S. Photochemical Reactivity of Perfluorooctanoic Acid (PFOA) in Conditions Representing Surface Water. *Science of the Total Environment* **2011**, *409* (16), 3043–3048.
(33) Bertucci, S.; Lova, P. Exploring Solar Energy Solutions for Per- and Polyfluoroalkyl Substances Degradation: Advancements and Future Directions in Photocatalytic Processes. *Solar RRL* **2024**, *8*(9), 2400116.
(34) Zheng, G.; Eick, S. M.; Salamova, A. Elevated Levels of Ultrashort- and Short-Chain Perfluoroalkyl Acids in US Homes and People. *Environmental Science and Technology* **2023**, *57* (42), 15782–15793.
(35) Wang, Y.; Zhang, P. Photocatalytic Decomposition of Perfluorooctanoic Acid (PFOA) by TiO 2 in the Presence of Oxalic Acid. *Journal of Hazardous Materials* **2011**, *192* (3), 1869–1875.
(36) Sansotera, M.; Persico, F.; Pirola, C.; Navarrini, W.; Di Michele, A.; Bianchi, C. L. Decomposition of Perfluorooctanoic Acid Photocatalyzed by Titanium Dioxide: Chemical Modification of the Catalyst Surface Induced by Fluoride Ions. *Applied Catalysis B* **2014**, *148*, 29–35.
(37) Buxton, G. V.; Greenstock, C. L.; Helman, W. P.; Ross, A. B. Critical Review of Rate Constants for Reactions of Hydrated Electrons, Hydrogen Atoms and Hydroxyl Radicals (·OH/·O− in Aqueous Solution. *Journal of Physical Chemistry* **1988**, *17* (2), 513–886.
(38) Javed, H.; Lyu, C.; Sun, R.; Zhang, D.; Alvarez, P. J. J. Discerning the Inefficacy of Hydroxyl Radicals during Perfluorooctanoic Acid Degradation. *Chemosphere* **2020**, *247*. *247*, 125883.
(39) Lymar, S. V; Schwarz, H. A.; Czapski, G. Medium Effects on Reactions of the Carbonate Radical with Thiocyanate, Iodide, and Ferrocyanide Ions. *Radiation Physics and Chemistry* **2000**, *59*(4), 387–392.
(40) Phan Thi, L. A.; Do, H. T.; Lee, Y. C.; Lo, S. L. Photochemical Decomposition of Perfluorooctanoic Acids in Aqueous Carbonate Solution with UV Irradiation. *Chemical Engineering Journal* **2013**, *221*, 258–263.
(41) Eberson, L. Electron-Transfer Reactions in Organic Chemistry. *Advances in Physical Organic Chemistry* **1982**, *78*, 79–185. https://doi.org/10.1016/S0065-3160(08)60139-2.

(42) Hori, H.; Yamamoto, A.; Hayakawa, E.; Taniyasu, S.; Yamashita, N.; Kutsuna, S.; Kiatagawa, H.; Arakawa, R. Efficient Decomposition of Environmentally Persistent Perfluorocarboxylic Acids by Use of Persulfate as a Photochemical Oxidant. *Environmental Science and Technology* **2005**, *39* (7), 2383–2388.
(43) Qian, Y.; Guo, X.; Zhang, Y.; Peng, Y.; Sun, P.; Huang, C. H.; Niu, J.; Zhou, X.; Crittenden, J. C. Perfluorooctanoic Acid Degradation Using UV-Persulfate Process: Modeling of the Degradation and Chlorate Formation. *Environmental Science and Technology* **2016**, *50* (2), 772–781.
(44) Wang, B. B.; Cao, M. H.; Tan, Z. J.; Wang, L. L.; Yuan, S. H.; Chen, J. Photochemical Decomposition of Perfluorodecanoic Acid in Aqueous Solution with VUV Light Irradiation. *Journal of Hazardous Materials* **2010**, *181* (1–3), 187–192.
(45) Bao, Y.; Deng, S.; Jiang, X.; Qu, Y.; He, Y.; Liu, L.; Chai, Q.; Mumtaz, M.; Huang, J.; Cagnetta, G.; Yu, G. Degradation of PFOA Substitute: GenX (HFPO-DA Ammonium Salt): Oxidation with UV/Persulfate or Reduction with UV/Sulfite? *Environmental Science and Technology*. **2018**, *52* (20), 11728–11734.
(46) Yang, S.; Cheng, J.; Sun, J.; Hu, Y.; Liang, X. Defluorination of Aqueous Perfluorooctanesulfonate by Activated Persulfate Oxidation. *PLoS One* **2013**, *8* (10), e74877.
(47) Fennell, B. D.; Mezyk, S. P.; McKay, G. Critical Review of UV-Advanced Reduction Processes for the Treatment of Chemical Contaminants in Water. *ACS Environmental Au* **2022**, *2*(3), 178–205.
(48) Park, H.; Vecitis, C. D.; Cheng, J.; Choi, W.; Mader, B. T.; Hoffmann, M. R. Reductive Defluorination of Aqueous Perfluorinated Alkyl Surfactants: Effects of Ionic Headgroup and Chain Length. *Journal of Physical Chemistry A* **2009**, *113* (4), 690–696.
(49) Liu, F.; Guan, X.; Xiao, F. Photodegradation of Per- and Polyfluoroalkyl Substances in Water: A Review of Fundamentals and Applications. *Journal of Hazardous Materials*. Elsevier B.V. October 5, **2022**, *439*, 129580.
(50) Bentel, M. J.; Yu, Y.; Xu, L.; Li, Z.; Wong, B. M.; Men, Y.; Liu, J. Defluorination of Per- and Polyfluoroalkyl Substances (PFASs) with Hydrated Electrons: Structural Dependence and Implications to PFAS Remediation and Management. *Environmental Science and Technology* **2019**, *53* (7), 3718–3728.
(51) Gu, Y.; Dong, W.; Luo, C.; Liu, T. Efficient Reductive Decomposition of Perfluorooctanesulfonate in a High Photon Flux UV/Sulfite System. *Environmental Science and Technology* **2016**, *50* (19), 10554–10561.
(52) Qu, Y.; Zhang, C.; Li, F.; Chen, J.; Zhou, Q. Photo-Reductive Defluorination of Perfluorooctanoic Acid in Water. *Water Research* **2010**, *44* (9), 2939–2947.
(53) Song, Z.; Tang, H.; Wang, N.; Zhu, L. Reductive Defluorination of Perfluorooctanoic Acid by Hydrated Electrons in a Sulfite-Mediated UV Photochemical System. *Journal of Hazardous Materials* **2013**, *262*, 332–338.
(54) Ren, Z.; Bergmann, U.; Leiviskä, T. Reductive Degradation of Perfluorooctanoic Acid in Complex Water Matrices by Using the UV/Sulfite Process. *Water Research* **2021**, *205*, 117676.
(55) Lee, Y. C.; Liu, Y. H.; Lin, J. C.; Hu, C. Y.; Kuo, J.; Lin, Y. C.; Lo, S. L. UV/Sulfite Reduction Kinetics of Perfluorobutane Sulfonic Acid (PFBS), Perfluorooctane Sulfonic Acid (PFOS) and Perfluorooctanoic Acid (PFOA). *Separation and Purification Technology* **2024**, *347*, 127505.
(56) Amador, C. K.; Vyas, S.; Strathmann, T. J. Kinetic Model for Predicting Perfluoroalkyl Acid Degradation During UV-Sulfite Treatment. *Environmental Science and Technology* **2024**, *58* (14), 6425–6434.
(57) Banayan Esfahani, E.; Asadi Zeidabadi, F.; Zhang, S.; Mohseni, M. Photo-Chemical/Catalytic Oxidative/Reductive Decomposition of per- and Poly-Fluoroalkyl Substances (PFAS), Decomposition Mechanisms and Effects of Key Factors: A Review. *Environmental Science: Water Research and Technology*, **2022**, *8*(4), 698–728.
(58) O'Connor, N.; Patch, D.; Noble, D.; Scott, J.; Koch, I.; Mumford, K. G.; Weber, K. Forever No More: Complete Mineralization of per- and Polyfluoroalkyl Substances (PFAS) Using an Optimized UV/Sulfite/Iodide System. *Science of the Total Environment* **2023**, *888*, 164137.
(59) Liu, Z.; Chen, Z.; Gao, J.; Yu, Y.; Men, Y.; Gu, C.; Liu, J. Accelerated Degradation of Perfluorosulfonates and Perfluorocarboxylates by UV/Sulfite + Iodide: Reaction Mechanisms and System Efficiencies. *Environmental Science and Technology* **2022**, *56* (6), 3699–3709.

(60) Tian, H.; Gao, J.; Li, H.; Boyd, S. A.; Gu, C. Complete Defluorination of Perfluorinated Compounds by Hydrated Electrons Generated from 3-Indole-Acetic-Acid in Organomodified Montmorillonite. *Scientific Reports* **2016**, *6*(1), 32949.
(61) Chen, Z.; Dong, R.; Wang, X.; Huang, L.; Qiu, L.; Zhang, M.; Mi, N.; Xu, M.; He, H.; Gu, C. Efficient Decomposition of Perfluoroalkyl Substances by Low Concentration Indole: New Insights into the Molecular Mechanisms. *Environmental Science and Technology* **2023**, *58*(7), 3530–3539.
(62) Jin, X.; Wang, Z.; Hong, R.; Chen, Z.; Wu, B.; Ding, S.; Zhu, W.; Lin, Y.; Gu, C. Supramolecular Assemblies of a Newly Developed Indole Derivative for Selective Adsorption and Photo-Destruction of Perfluoroalkyl Substances. *Water Research* **2022**, *225*, 119147.
(63) Wang, Z.; Jin, X.; Hong, R.; Wang, X.; Chen, Z.; Gao, G.; He, H.; Liu, J.; Gu, C. New Indole Derivative Heterogeneous System for the Synergistic Reduction and Oxidation of Various Per-/Polyfluoroalkyl Substances: Insights into the Degradation/Defluorination Mechanism. *Environmental Science and Technology* **2023**, *57* (50), 21459–21469.
(64) Schneider, J.; Matsuoka, M.; Takeuchi, M.; Zhang, J.; Horiuchi, Y.; Anpo, M.; Bahnemann, D. W. Understanding TiO2 photocatalysis: Mechanisms and Materials. *Chemical Reviews* **2014**, *114* (19), 9919–9986.
(65) Song, Z.; Dong, X.; Wang, N.; Zhu, L.; Luo, Z.; Fang, J.; Xiong, C. Efficient Photocatalytic Defluorination of Perfluorooctanoic Acid over BiOCl Nanosheets via a Hole Direct Oxidation Mechanism. *Chemical Engineering Journal* **2017**, *317*, 925–934.
(66) Wu, Y.; Hu, Y.; Han, M.; Ouyang, Y.; Xia, L.; Huang, X.; Hu, Z.; Li, C. Mechanism Insights into the Facet-Dependent Photocatalytic Degradation of Perfluorooctanoic Acid on BiOCl Nanosheets. *Chemical Engineering Journal* **2021**, *425*, 130672.
(67) Xu, B.; Zhou, J. L.; Altaee, A.; Ahmed, M. B.; Johir, M. A. H.; Ren, J.; Li, X. Improved Photocatalysis of Perfluorooctanoic Acid in Water and Wastewater by Ga2O3/UV System Assisted by Peroxymonosulfate. *Chemosphere* **2020**, *239*, 124722.
(68) Khan, H.; Gar Alalm, M.; Lalonde-Lavoie, M.; Ordonez, M. F.; Sartirana, M.; Giordana, A.; Cerrato, G.; Bianchi, C. L.; Boffito, D. C. Photocatalytic Degradation of NOx and Ethanol in the Gas Phase by Spray Dried Ce-TiO2. *Journal of Environmental Chemical Engineering* **2021**, *9* (6), 106813.
(69) Li, F.; Wei, Z.; He, K.; Blaney, L.; Cheng, X.; Xu, T.; Liu, W.; Zhao, D. A Concentrate-and-Destroy Technique for Degradation of Perfluorooctanoic Acid in Water Using a New Adsorptive Photocatalyst. *Water Res.* **2020**, *185*, 116219.
(70) Yang, Y.; Ji, W.; Li, X.; Zheng, Z.; Bi, F.; Yang, M.; Xu, J.; Zhang, X. Insights into the Degradation Mechanism of Perfluorooctanoic Acid under Visible-Light Irradiation through Fabricating Flower-Shaped Bi5O7I/ZnO n-n Heterojunction Microspheres. *Chemical Engineering Journal* **2021**, *420*, 129934.
(71) Lin, H.; Niu, J.; Ding, S.; Zhang, L. Electrochemical Degradation of Perfluorooctanoic Acid (PFOA) by Ti/SnO2-Sb, Ti/SnO2-Sb/PbO2 and Ti/SnO2-Sb/MnO2 Anodes. *Water Research* **2012**, *46* (7), 2281–2289.
(72) Song, Z.; He, J.; Kouzehkanan, S. M. T.; Oh, T. S.; Olshansky, Y.; Duin, E. C.; Carroll, K. C.; Wang, D. Enhanced Sorption and Destruction of PFAS by Biochar-Enabled Advanced Reduction Process. *Chemosphere* **2024**, *363*, 142760.
(73) Xu, T.; Zhu, Y.; Duan, J.; Xia, Y.; Tong, T.; Zhang, L.; Zhao, D. Enhanced Photocatalytic Degradation of Perfluorooctanoic Acid Using Carbon-Modified Bismuth Phosphate Composite: Effectiveness, Material Synergy and Roles of Carbon. *Chemical Engineering Journal* **2020**, *395*, 124991.
(74) Boonchata, P.; Boontanon, N.; Jindal, V.; Aung, H. K. Z. Z.; Visvanathan, C.; Fujii, S.; Boontanon, S. K. Photocatalytic Degradation of Perfluorooctane Sulfonic Acid and Perfluorooctanoic Acid Using Titanium Dioxide/Graphene Oxide Nanocomposite Immobilized on Polyvinyl Alcohol Film. *Case Studies in Chemical and Environmental Engineering* **2024**, *10*, 100862.
(75) Panchangam, S. C.; Yellatur, C. S.; Yang, J. S.; Loka, S. S.; Lin, A. Y. C.; Vemula, V. Facile Fabrication of TiO2-Graphene Nanocomposites (TGNCs) for the Efficient Photocatalytic Oxidation of Perfluorooctanoic Acid (PFOA). *Journal of Environmental Chemical Engineering* **2018**, *6* (5), 6359–6369.
(76) Arana Juve, J. M.; Li, F.; Zhu, Y.; Liu, W.; Ottosen, L. D. M.; Zhao, D.; Wei, Z. Concentrate and Degrade PFOA with a Photo-Regenerable Composite of In-Doped TNTs@AC. *Chemosphere* **2022**, *300*, 134495.

(77) Zhu, Y.; Leary, R. N.; Xu, T.; He, K.; Blaney, L.; Hao, X.; Zhao, D. Photocatalytic Degradation of PFAS under Field Water Matrix Conditions Using an Adsorptive Photocatalyst. *Water Research X* **2026**, *30*, 100485.

(78) García-Castrillo, M.; Barandika, G.; Lizundia, E. Low Environmental Impact Magnetic Chitosan and Chitin Cryogels for PFAS Remediation. *Advanced Functional Materials* **2024**, *36*(46), 2405298.

(79) KARA, I. T.; KIYAK, B.; GUNES, N. C.; YUCEL, S. Life Cycle Assessment of Aerogels: A Critical Review. *Journal of Sol-Gel Science and Technology* **2024,** *111*(2), 618–649.

(80) Roman, J. B.; Kemperman, A. J. B.; van der Meer, W. G. J.; Wood, J. A. Assessing the Viability of Bio-Based Adsorbents for PFAS Removal. *Chemical Engineering Science* **2025**, *306*, 121215.

(81) Shen, L.; Worrell, E.; Patel, M. K. Environmental Impact Assessment of Man-Made Cellulose Fibres. *Resources, Conservation and Recycling* **2010**, *55* (2), 260–274.

(82) Moretti, C.; Corona, B.; Hoefnagels, R.; Vural-Gürsel, I.; Gosselink, R.; Junginger, M. Review of Life Cycle Assessments of Lignin and Derived Products: Lessons Learned. *Science of the total environment* **2021**, *770*, 144656.

(83) EU consortium PortableCrac. LCA & LCCA of Acquiring Virgin Ac and of Thermal Regeneration **2018**. https://doi.org/10.3030/768905.

(84) Kim, M. H.; Jeong, I. T.; Park, S. B.; Kim, J. W. Analysis of Environmental Impact of Activated Carbon Production from Wood Waste. *Environmental Engineering Research* **2018**, *24* (1), 117–126.

(85) León, M.; Silva, J.; Carrasco, S.; Barrientos, N. Design, Cost Estimation and Sensitivity Analysis for a Production Process of Activated Carbon Fromwaste Nutshells by Physical Activation. *Processes* **2020**, *8* (8), 945.

(86) Luo, Y.; Razzaq, M. E. A.; Qu, W.; Mohammed, A. A. B. A.; Aui, A.; Zobeiri, H.; Wright, M. M.; Wang, X.; Bai, X. Introducing Thermo-Mechanochemistry of Lignin Enabled the Production of High-Quality Low-Cost Carbon Fiber. *Green Chemistry* **2024**, *26* (6), 3281–3300.

(87) Arzate, S.; Pfister, S.; Oberschelp, C.; Sánchez-Pérez, J. A. Environmental Impacts of an Advanced Oxidation Process as Tertiary Treatment in a Wastewater Treatment Plant. *Science of the Total Environment* **2019**, *694*, 133572.

(88) Cabezuelo, O.; Diego-López, A.; Marin, M. L.; Bosca, F. Mineralization of Pollutants in Water at Neutral PH through a Heterogeneous Photo-Fenton-like Process Generated by SiO2@TiO2@Fe3O4 in the Presence of PMS. *Journal of Environmental Chemical Engineering* **2025**, *13*(3), 116220.

(89) Brüninghoff, R.; Van Duijne, A. K.; Braakhuis, L.; Saha, P.; Jeremiasse, A. W.; Mei, B.; Mul, G. Comparative Analysis of Photocatalytic and Electrochemical Degradation of 4-Ethylphenol in Saline Conditions. *Environmental Science and Technology* **2019**, *53* (15), 8725–8735.

(90) Babbit, C. W.; Stokke, J. M.; Mazyck, D. W.; Lindner, A. S. Design-Based Life Cycle Assessment of Hazardous Air Pollutant Control Options at Pulp and Paper Mills: A Comparison of Thermal Oxidation to Photocatalytic Oxidation and Biofiltration. *Journal of Chemical Technology and Biotechnology* **2009**, *84* (5), 725–737.

(91) Pesqueira, J. F. J. R.; Pereira, M. F. R.; Silva, A. M. T. A Life Cycle Assessment of Solar-Based Treatments (H2O2, TiO2 Photocatalysis, Circumneutral Photo-Fenton) for the Removal of Organic Micropollutants. *Science of the Total Environment* **2021**, *761*, 143258.

(92) Cabezuelo, O.; Ponce-Gonzalez, L. N.; Marin, M. L.; Bosca, F. A Highly Efficient Supported TiO2 Photocatalyst for Wastewater Remediation in Continuous Flow. *Applied Materials Today* **2023**, *35*, 101947.

(93) Cabezuelo, O.; Diego-Lopez, A.; Atienzar, P.; Luisa Marin, M.; Bosca, F. Optimizing the Use of Light in Supported TiO2 Photocatalysts: Relevance of the Shell Thickness. *Journal of Photochemistry and Photobiology A: Chemistry* **2023**, *444*, 114917.

(94) Singh, S.; Suthar, G.; Bhushan Gupta, A.; Bezbaruah, A. N. Machine Learning Approach for Predicting Perfluorooctanesulfonate Rejection in Efficient Nanofiltration Treatment and Removal. *ACS ES&T Water* **2025**, *5*(3), 1216–1228.

(95) Jeong, N.; Park, S.; Mahajan, S.; Zhou, J.; Blotevogel, J.; Li, Y.; Tong, T.; Chen, Y. Elucidating Governing Factors of PFAS Removal by Polyamide Membranes Using Machine Learning and Molecular Simulations. *Nature Communications* **2024**, *15*(1), 10918.

(96) Hosseinzadeh, A.; Zhou, J. L.; Zyaie, J.; AlZainati, N.; Ibrar, I.; Altaee, A. Machine Learning-Based Modeling and Analysis of PFOS Removal from Contaminated Water by Nanofiltration Process. *Separation and Purification Technology* **2022**, *289*, 120775.
(97) Yuan, S.; Wang, X.; Jiang, Z.; Zhang, H.; Yuan, S. Contribution of Air-Water Interface in Removing PFAS from Drinking Water: Adsorption, Stability, Interaction and Machine Learning Studies. *Water research* **2023**, *236*, 119947.
(98) Ersan, G.; Lukman, A. Can Machine Learning Algorithms Enhance the Prediction Accuracy of Linear Solvation Energy Relationship Models for Polyfluoroalkyl Substances Adsorption by Activated Carbons in Complex Water Matrices? *ACS ES&T Water* **2024**, *5*(1), 479–487.
(99) Kazi, M. K.; Varghese, S.; Sarker, N.; Aich, N.; Gadhamshetty, V. Advancing PFAS Remediation through Physics-Based Modeling of 2D Materials: Recent Progress, Challenges, and Opportunities. *Industrial and Engineering Chemistry Research* **2025**, 1894–1906.
(100) Nasrollahpour, S.; Tanhadoust, A.; Pulicharla, R.; Brar, S. K. Long-Chain Perfluoroalkyl Carboxylic Acids Removal by Biochar: Experimental Study and Uncertainty Based Data-Driven Predictive Model. *iScience* **2024**, *27* (11).
(101) Patel, H. V.; Green, J.; Park, H.-S. (John); Luster-Teasley Pass, S.; Zhao, R. Quantifying Multidimensional Effects of Physicochemical Parameters on PFAS Adsorption Using a Hybrid Response Surface Methodology-Machine Learning Approach. **2025** https://doi.org/10.26434/chemrxiv-2025-3wl26
(102) Zhang, J.; Fu, K.; Zhong, S.; & Luo, J; Data-driven insights into resin screening for targeted per-and polyfluoroalkyl substances removal using machine learning. *Environmental Science & Technology* **2025**, *59*(7), 3603–3612.
(103) Navidpour, A. H.; Hosseinzadeh, A.; Huang, Z.; Li, D.; Zhou, J. L. Application of Machine Learning Algorithms in Predicting the Photocatalytic Degradation of Perfluorooctanoic Acid. *Catalysis Reviews* **2024**, *66*(2), 687-712.
(104) Cao, H.; Peng, J.; Zhou, Z.; Sun, Y.; Wang, Y.; Liang, Y. Insight into the Defluorination Ability of Per- and Polyfluoroalkyl Substances Based on Machine Learning and Quantum Chemical Computations. *Science of the Total Environment* **2022**, *807*, 151018.
(105) Dashti, A.; Navidpour, A. H.; Amirkhani, F.; Zhou, J. L.; Altaee, A. Application of Machine Learning Models to Improve the Prediction of Pesticide Photodegradation in Water by ZnO-Based Photocatalysts. *Chemosphere* **2024**, *362*, 142792.
(106) Raza, A.; Bardhan, S.; Xu, L.; Yamijala, S. S. R. K. C.; Lian, C.; Kwon, H.; Wong, B. M. A Machine Learning Approach for Predicting Defluorination of Per- and Polyfluoroalkyl Substances (PFAS) for Their Efficient Treatment and Removal. *Environmental Science & Technology Letters* **2019**, *6* (10), 624–629. https://doi.org/10.1021/acs.estlett.9b00476.
(107) Giri, R. R.; Ozaki, H.; Morigaki, T.; Taniguchi, S.; Takanami, R. UV Photolysis of Perfluorooctanoic Acid (PFOA) in Dilute Aqueous Solution. *Water Science and Technology* **2011**, *63* (2), 276–282.
(108) Chong, M. C.; Zhou, Q.; Xu, J.; Wu, Z.; Zhu, E.; Li, W.; Zhang, L.; Guo, Y.; Zhu, Y. Complete Defluorination of PFASs via Photocatalytic Reduction in Water. *Nature Communications* **2026**, *17* (1).
(109) Fernando, E. Y.; Sarkar, D.; Rodwihok, C.; Satpathy, A.; Zhang, J.; Rahmati, R.; Datta, R.; Christodoulatos, C.; Boufadel, M.; Larson, S.; Zhang, Z. Unrefined and Milled Ilmenite as a Cost-Effective Photocatalyst for UV-Assisted Destruction and Mineralization of PFAS. *Materials* **2024**, *17* (15), 3801.
(110) Yang, H.; Park, S. J.; Lee, C. G. Enhanced Removal of Perfluoroalkyl Substances Using MMO-TiO2 Visible Light Photocatalyst. *Alexandria Engineering Journal* **2024**, *87*, 31–38.
(111) Wang, Z.; Li, M.; Cao, W.; Liu, Z.; Kong, D.; Jiang, W. Efficient Photocatalytic Degradation of Perfluorooctanoic Acid by Bismuth Nanoparticle Modified Titanium Dioxide. *Science of the Total Environment* **2024**, *927*, 172028.
(112) Chen, A.; Ji, H.; Xu, Z.; Wang, Z.; Xie, H.; Zhang, L. Unique S-Scheme Structures in Electrostatic Self-Assembled ReS2-TiO2 for High-Efficiency Perfluorooctanoic Acid Removal. *J. Colloid Interface Science* **2025**, *681*, 63–70.
(113) Nejatpour, M.; Ünsür, A. M.; Yılmaz, B.; Gül, M.; Ozden, B.; Barisci, S.; Dükkancı, M. Enhanced Photodegradation of Perfluorocarboxylic Acids (PFCAs) Using Carbon Quantum Dots (CQDs) Doped

TiO2 Photocatalysts: A Comparative Study between Exfoliated Graphite and Mussel Shell-Derived CQDs. *J. Environmental Chemical Engineering* **2025**, *13* (1), 115382.
(114) Ünsür, A. M.; Nejatpour, M.; Dükkancı, M.; Ozden, B.; Barisci, S. Enhanced Photocatalytic Degradation of PFOA and Short-Chain PFCAs Coupled with Hydrogen Production via Olive Kernel Biomass-Based CQDs-Doped TiO2 Composites. *Separation and Purification Technology* **2026**, *385*, 136366.
(115) Qian, L.; Zhao, H.; Schierz, A.; Mackenzie, K.; Georgi, A. A Deep Insight into Perfluorooctanoic Acid Photodegradation Using Metal Ion-Exchanged Zeolites. *ACS ES and T Engineering* **2024**, *4* (3), 748–757.
(116) Li, X.; Xiao, X.; Zhang, S.; Teng, X.; Wang, Z.; Qu, R. Self-Regenerable Zeolite Beta(35) for Efficient Adsorption and Photocatalytic Degradation of PFOA under Simulated Solar Irradiation. *Water Research* **2026**, *288*, 124645.
(117) Liu, X.; Xu, B.; Duan, X.; Hao, Q.; Wei, W.; Wang, S.; Ni, B. J. Facile Preparation of Hydrophilic In2O3nanospheres and Rods with Improved Performances for Photocatalytic Degradation of PFOA. *Environmental Science: Nano* **2021**, *8* (4), 1010–1018.
(118) Zhang, L.; Ni, B. J.; Liu, X.; Chen, Z.; Tian, K.; Zhu, F.; Hao, D.; Cheng, D.; Wei, W. Fe3+ Promoted the Photocatalytic Defluorination of Perfluorooctanoic Acid (PFOA) over In2O3. *ACS ES and T Water* **2021**, *1* (11), 2431-2439.
(119) Wu, Y.; Li, Y.; Fang, C.; Li, C. Highly Efficient Degradation of Perfluorooctanoic Acid over a MnO x - Modified Oxygen-Vacancy-Rich In 2 O 3 Photocatalyst. *ChemCatChem* **2019**, *11* (9), 2297–2303.
(120) Liu, J.; Guo, C.; Wu, N.; Li, C.; Qu, R.; Wang, Z.; Jin, R.; Qiao, Y.; He, Z.; Lu, J.; Feng, X.; Zhang, Y.; Wang, A.; Gao, J. Efficient Photocatalytic Degradation of PFOA in N-Doped In2O3/Simulated Sunlight Irradiation System and Its Mechanism. *Chemical Engineering Journal* **2022**, *435*, 134627.
(121) Zhang, W.; Efstathiadis, H.; Li, L.; Liang, Y. Environmental Factors Affecting Degradation of Perfluorooctanoic Acid (PFOA) by In2O3 Nanoparticles. *Journal of Environmental Sciences* **2020**, *93*, 48–56.
(122) Wang, S.; Wang, S.; Tang, R.; Liu, Y.; Chen, H. Removal of Mixed Pollutants of Perfluorooctanoic Acid and 2,4,6-Trichlorophenol via Adsorption-Photocatalysis Using Ga2O3-Bi4O7 Combining with Fluorine-Doped Covalent Triazine Framework: Role of Different Active Species. *Journal of Colloid and Interface Science* **2024**, *676*, 959–973.
(123) Huang, J.; Zou, Y.; Ling, L. Enhanced Selective Photocatalytic Reduction and Oxidation of Perfluorooctanoic Acid on Bi/Bi5O7I Decorated with Poly (Triazine Imide). *Journal of Hazardous Materials* **2024**, *480*, 136257.
